# Streamlining Computational Fragment-Based Drug Discovery through Evolutionary Optimization Informed by Ligand-Based Virtual Prescreening

**DOI:** 10.1021/acs.jcim.4c00234

**Published:** 2024-05-02

**Authors:** Rohan Chandraghatgi, Hai-Feng Ji, Gail L. Rosen, Bahrad A. Sokhansanj

**Affiliations:** † Department of Chemistry, Drexel University, Philadelphia, Pennsylvania 19104, United States; ‡ Department of Electrical & Computer Engineering, Drexel University, Philadelphia, Pennsylvania 19104, United States; § Department of Biology, Drexel University, Philadelphia, Pennsylvania 19104, United States

## Abstract

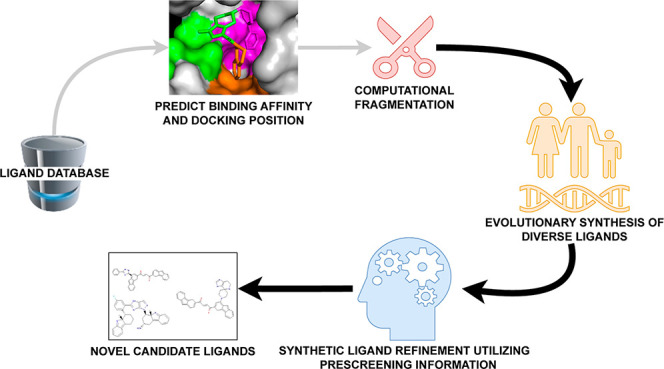

Recent advances in computational methods provide the promise of dramatically accelerating drug discovery. While mathematical modeling and machine learning have become vital in predicting drug–target interactions and properties, there is untapped potential in computational drug discovery due to the vast and complex chemical space. This paper builds on our recently published computational fragment-based drug discovery (FBDD) method called fragment databases from screened ligand drug discovery (FDSL-DD). FDSL-DD uses in silico screening to identify ligands from a vast library, fragmenting them while attaching specific attributes based on predicted binding affinity and interaction with the target subdomain. In this paper, we further propose a two-stage optimization method that utilizes the information from prescreening to optimize computational ligand synthesis. We hypothesize that using prescreening information for optimization shrinks the search space and focuses on promising regions, thereby improving the optimization for candidate ligands. The first optimization stage assembles these fragments into larger compounds using genetic algorithms, followed by a second stage of iterative refinement to produce compounds with enhanced bioactivity. To demonstrate broad applicability, the methodology is demonstrated on three diverse protein targets found in human solid cancers, bacterial antimicrobial resistance, and the SARS-CoV-2 virus. Combined, the proposed FDSL-DD and a two-stage optimization approach yield high-affinity ligand candidates more efficiently than other state-of-the-art computational FBDD methods. We further show that a multiobjective optimization method accounting for drug-likeness can still produce potential candidate ligands with a high binding affinity. Overall, the results demonstrate that integrating detailed chemical information with a constrained search framework can markedly optimize the initial drug discovery process, offering a more precise and efficient route to developing new therapeutics.

## 1 Introduction

Advances in computational methodologies have revolutionized drug discovery. Mathematical modeling and machine learning techniques have emerged as vital tools to predict drug–target interactions and drug properties.^1−7^ However, computation has yet to be employed to its full potential in the drug discovery process. One key area for innovation is computational drug discovery.^8−10^ As the low-hanging fruit for novel drugs has been harvested, it is increasingly difficult to find promising lead compounds.^11−13^ The now-conventional approach to drug design relies on high-throughput screening of large compound libraries, which is often time-consuming and resource intensive.^14−17^ Computational drug discovery has the potential to leverage the power of in silico screening by using machine learning and optimization techniques to predict the bioactivity of potential compounds and thereby accelerate drug discovery.

Recently, “fragment-based drug discovery” (or “design”) (FBDD) has emerged as a potentially promising approach;^18−23^ it is unlike other drug design strategies (i.e., structure-based drug discovery, SBDD, or ligand-based drug discovery, LBDD), which usually involve designing or testing full-sized drug molecules or identifying smaller chemical fragments that bind effectively to target biomolecules. These small fragments serve as building blocks that can be grown, linked, or merged to create new drug molecules. The previously proposed FBDD offers a flexible and efficient way to explore the vast potential space of drug-like molecules. However, despite the potential advantages of FBDD, a significant challenge remains: the scale of the chemical space to be explored. Given the enormous diversity of possible chemical fragments and the ways in which they can be combined, the number of potential drug candidates is effectively infinite. This massive combinatorial problem can become a stumbling block, slowing the drug discovery process and making it difficult to identify promising candidates. This paper proposes a solution to this challenge, centered on the computational generation of potential drug molecules.

Computational de novo drug design involves the use of techniques such as genetic algorithms,^24−29^ reinforcement learning, including deep reinforcement learning,^30−34^ generative deep learning models,^35−41^ or other deep learning methods, e.g., graph transformers,^42,43^ models that blend deep learning and evolutionary algorithms,^26,44,45^ and string-based transformers (i.e., operating on a simplified molecular-input line-entry system (SMILES) string representation of molecules).^46,47^ The algorithms “computationally synthesize” novel drug molecules, either by starting from scratch and adding atoms to form a novel molecule or by modifying or adding atoms to an existing chemical structure (“scaffold”). The result is the creation of novel molecules by (a) simulating chemical modifications that optimize for the single objective of improving binding efficiency to a target or (b) multiobjective optimization including drug-likeness objectives, e.g., solubility and other drug-likeness factors.^25,48−55^

In computational FBDD, various in silico computational techniques are utilized to construct fragment libraries for FBDD. The conventional approach to computational FBDD involves either computationally fragmentizing a compound (ligand) library or self-generating fragments using computational techniques, followed by computationally docking target fragments to a protein binding pocket and computationally “growing” or synthesizing a candidate ligand by modifying the fragment within that pocket.^56^ Methods like FastGrow emphasize identifying fragment growth points rather than the specifics of fragment expansion, often comparing to other structural docking tools.^57^ In the realm of docking, ultralarge-scale docking techniques, such as those by Lyu et al., identify potential molecules based on docking scores, with a breadth possibly surpassing human intuition.^58^ On a similar note, Allen et al. present iterative fragment growth relying on docking scores, yet distinctively using prescreening information to navigate their search.^59^ The advent of “deep evolutionary learning” for FBDD introduced the employment of a latent space grounded in SMILES, as seen in methods like FragVAE, which incorporates evolutionary operators and data augmentation in the process.^45^ Subsequent techniques, such as Podda et al.’s encoder-decoder generative model, also employ the SMILES structure to produce fragments.^60^ More advanced strategies integrate graph-based and evolutionary operators on a molecule’s latent representation, focusing on multiobjective optimization.^54^ While some approaches start from a template and deploy generative methods for modification based on structure–activity relationship (SAR),^61^ others like Cortés-Cabrera et al. use a fragment approach, progressively constructing a molecule and utilizing the ligand efficiency index (LEI) for guidance.^62^ A notable method introduced by Kerstjens and De Winter applies new genetic operators to fragment- and graph-based evolutionary designs, emphasizing atom compatibility rules.^63^ Many of these methods, including those that utilize reaction rules or grow rules, emphasize the growth of fragments within 3D binding pockets, a feature that resembles this research’s approach.^64^ Advanced techniques, such as those by Tang et al., combine deep reinforcement learning with chemistry to sculpt fragment libraries.^65^ In the wake of these innovations, researchers are also capitalizing on SMILES using transformers to decorate scaffolds, iterating on their multiobjective techniques as seen in the advancements from Liu et al.^42,66^

Despite the sophistication of current algorithms, the vastness of the exploration space and the plethora of potential optima remain daunting challenges. Leveraging either heuristic (evolutionary) approaches or learning techniques, these methods aim to identify superior optima. However, given the expansive nature of the chemical space, any strategy will fail to be a universally optimal solution to all possible problems and chemical configurations.^67^ In addition to the limits on heuristic optimization methods, deep learning methods are limited because training typically probes only a minute subspace of the chemical structure landscape. The pivotal question that emerges is Can we harness problem-specific chemical information to effectively curtail the massively complex search space of potential chemical structures?

To reduce the combinatorial search space and achieve more targeted drug designs, we leverage a pipeline for computational FBDD recently developed by our group called fragments from ligands drug discovery (FDSL-DD).^68^ The FDSL-DD pipeline involves an initial in silico screening step of a large ligand database against a protein target, utilizing computational docking software, e.g., Autodock VINA. The ligands are then computationally fragmentized, and the fragments are assigned information (i.e., fragment attributes) based on the predicted binding affinity (docking score) and amino acids that are predicted to interact with atoms in the computational fragment. The information output from the FDSL-DD pipeline can then be used to resynthesize the fragments in novel combinations and generate synthetic ligands that could form the basis for potential candidate lead compounds. The FDSL-DD approach thereby contrasts with conventional computational FDBB, which, even when based on predetermined ligand libraries, does not retain information about protein–ligand binding based on initial virtual library screening.

**Figure 1:**
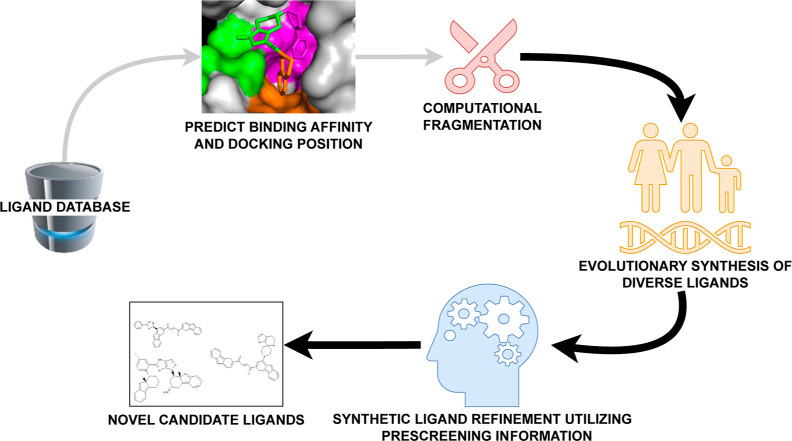
Proposed integrated FDSL-DD and optimization method for computational drug design for a specific protein target begins with a library of ligands, prescreened by docking them with Autodock VINA, computationally fragmented, and are assigned binding affinities and other attributes of the parent ligands computed in the prescreening process, as previously described.^68^ The fragments are recombined using an evolutionary algorithm informed by parent ligand attributes, which results in the creation of both a diverse computational ligand population and one that has more optimal characteristics. The ligands output from the first optimization stage are then refined through an iterative optimization that also utilizes parent ligand attributes as well as information about fragment position in the binding pocket, to ultimately generate a population of novel candidate ligands for further evaluation and validation. (The components connected with gray lines are fully described in our previous work,^68^ while the components connected with solid lines are the subject of this paper.).

Given the challenge of searching a huge combinatorial search space to find optimal candidate ligands for a given receptor, we hypothesize that using prescreening information for optimization will both shrink the search space for potential candidate ligands and focus on promising regions, thereby making the search for ligands both faster and more effective. Specifically, the optimization process will be focused on the likely most optimal regions of the search space since the starting fragments are from ligands that already have superior bioactivity, and the process is less likely to get trapped in suboptimal regions since the search space is effectively smaller. Accordingly, we build on the FDSL-DD framework introduced in Wilson et al.^68^ by creating a comprehensive computational drug design methodology. We propose and demonstrate two stages of optimization, which are based on applying the fragment structures as well as associated fragment attributes derived from ligand prescreening. Figure 1 shows a high-level schematic of the proposed approach. The two optimization stages proceed as follows: (1) evolutionary optimization uses principles of natural selection and genetic variation in a computational method for strategically guiding the assembly of synthetic fragments to generate larger compounds. (2) Iterative optimization refines the resulting compounds by adding small fragments to improve bioactivity. At both stages, the fragment information obtained from the FDSL-DD pipeline narrows the vast chemical space by imposing constraints that limit the search to areas with higher potential for success. Using fragment attributes will both reduce the combinatorial size of the search space and focus the search for ligands on potentially more favorable parts of the space. The resulting computational drug design process not only becomes more efficient but also more likely to yield compounds with high binding affinities and desirable drug-like properties. In our previous work, we looked for the highest binding affinity combinations of fragments from the highest binding affinity prescreened ligands but did not design an optimization methodology based on fragment attributes.

In this paper, we evaluate the proposed two-stage optimization on the initial prescreening that was performed in our previous work^68^ on three distinct protein targets found in different kinds of organisms, i.e., human, bacterial, and viral, and which are in turn implicated in very different kinds of diseases and contexts: (1) tumor necrosis factor-alpha-induced protein 8-like 2 (TIPE2), a transport protein that can induce leukocyte polarization, sustaining chronic inflammation and ultimately supporting solid cancer tumorigenesis;^69^ TIPE2 inhibition would provide a therapeutic option for solid tumor cancers. (2) Bacterial protein RelA, which plays a role in detecting amino acid starvation by activating a stringent response in bacteria that leads to persister cell formation, thus providing a potential inhibitory target to allow antibiotics to eradicate bacteria in biofilms.^70^ (3) The receptor binding domain (RBD) of the S1 subunit of the spike protein (S-protein) of severe acute respiratory syndrome coronavirus 2 (SARS-CoV-2) and the SARS-CoV-2 spike protein receptor binding domain (RBD), which binds to human angiotensin-converting enzyme (ACE-2), thereby facilitating viral entry and representing a potential target for antiviral therapeutics for COVID-19.^71^

The computational studies herein demonstrate the potential of the methods to significantly enhance the efficiency and effectiveness of computational drug design. First, we assess the utility of information about fragments obtained through the initial virtual screening step in FDSL-DD by showing that FDSL-DD with two-stage optimization results can computationally generate candidate ligands that have high predicting binding affinity, with substantially improved performance as compared to not using a virtual screening step (i.e., the “naive” approach of conventional computational FBDD). Second, we compare computational ligands generated by FDSL-DD and two-stage optimization to those generated by highly cited and well-documented conventional FBDD methods: (1) AutoGrow,^27,72^ which utilizes genetic algorithms, like the first-stage optimization of our method; and (2) DeepFrag,^73,74^ which utilizes deep learning to optimize computational fragment selection and growth based on characteristics of the protein binding pocket, which contrasts to the use of virtual ligand screening based on specific protein targets in our approach. We have created an open-source Python software to implement the algorithms presented in this paper and made it freely available for public noncommercial use. The software implementation has been optimized to run on multithreaded CPUs, and it can scalably run on very large ligand screening outputs in a multinode high-performance computing environment. The source code is available at https://github.com/EESI/FDSL_Evo.

## 2 Methods

### 2.1 Ligand Prescreening and Fragmentation Pipeline

Figure 1 shows the complete proposed computational drug design workflow, which begins with prescreening of a ligand library with protein targets. The prescreening pipeline portion of the workflow was applied to the protein targets shown in this paper in our prior publication.^68^ Briefly, the crystal structures of the protein files, TIPE2 (PDB ID: TIPE2), RelA (PDB ID: 5IQR), and S-protein (PDB ID: 6M0J), were retrieved from the RCSB Protein Data Bank. The structures were preprocessed by removing waters, cocrystallized proteins, and cocrystallized atoms. The S-glycoprotein was further run through a restriction minimization process utilizing Schrodinger docking suits protein preparation, which allows side chains to be placed in the most energetically favorable conformation. Protein structures were prepared in AutoDockTools-1.5.6,^75^ including the addition of polar hydrogens and calculation of Gasteiger charges. Ligands from an Enamine Ltd. “Drug-like” library consisting of around 250,000 molecules were retrieved and optimized using OpenBabel,^76^ generating 3D structures, adding charges, and minimizing with the MMFF94 force field.

High-throughput molecular docking calculations of mass libraries were performed using AutoDock Vina 1.2.3^77^ on Drexel University Research Computing Facility’s Picotte high-performance computing cluster of Intel Xeon Platinum 8268 CPUs. As required for Autodock VINA, grid boxes for ligand docking were generated for each protein-targeting known binding site. The grid box for RelA is centered at *x* = 297.894, *y* = 163.593, and *z* = 219.301 with dimensions of 25.000 Å. For the S-protein, the box of dimensions 22.000 Å × 42.000 Å × 22.000 Å was centered at *x* = −27.878, *y* = 25.205, and *z* = 5.514. TIPE2 has a particularly large binding cavity; therefore, 4 grid boxes were generated to span the pocket entrance, thereby occluding the cavity. Consequently, docking with TIPE2 generated 4 times the output, as every ligand was docked in each quadrant grid box. All grid box quadrants are of 12.000 Å dimensions with quadrant 1 centered at *x* = 60.677, *y* = 5.646, and *z* = 17.000; quadrant 2 centered at *x* = 62.636, *y* = 11.365, and *z* = 19.959; quadrant 3 centered at *x* = 68.067, *y* = 10.738, and *z* = 18.594; and quadrant 4 centered at *x* = 67.024, *y* = 5.362, and *z* = 17.113.

The lowest binding affinity bound ligand structure output by Autodock VINA is extracted and associated with its binding affinity value. The docked ligand structure’s PDBQT-format file is input to the protein ligand interaction profiler (PLIP).^78^

The ligand is also broken into fragments using BRICS,^79^ an algorithm designed to break bonds in a chemically realistic manner. BRICS fragmentation rules specifically target bonds in organic molecules that are considered synthetically feasible for breaking in retrosynthesis, which also helps retain essential pharmacophoric elements. BRICS rules primarily focus on cleaving bonds in certain functional groups and linkages. For instance, they target amide, ester, and ether linkages, which are common in drug-like molecules, allowing cleavage of the molecule at these points. The rules also include breaking of carbon-heteroatom bonds, especially in cases where the heteroatom is part of a ring structure or a key functional group. The BRICS fragmentation algorithm is implemented in Python 3.8 using the RDKit open source chemoinformatics software package, available at http://www.rdkit.org.

The resulting fragments are then associated with their “parent” ligands (the ligands that were fragmentized). Many fragments will have multiple parent ligands, i.e., they would have appeared as fragments of multiple ligands. The location of the fragment in the binding pocket is then identified for each parent ligand by finding the maximum common substructure (MCS) between the fragment and ligand.^80^ Each distinct fragment-subregion combination is then stored, with the fragment stored in a SMILES format^81^ along with the median binding affinity, the protein residues with bonds identified by PLIP, and the frequency it is found at in the prescreened ligand population.

### 2.2 Genetic Algorithm (Phase 1 of Fragment Synthesis)

The genetic algorithm requires the target receptor’s PDB (Protein Data Bank) file, an Autodock VINA configuration file (as described in the previous subsection for ligand prescreening), and the output of the fragmentation and analysis pipeline described in the preceding subsection. The resulting table is sorted based on the binding affinity to the receptor without considering target residues (i.e., the overall median binding affinity for each fragment). In the current version of the code, BRICS fragments are required, although some modification can make it compatible with any fragmentation protocol.

### 2.3 Genetic Algorithm Overview

An individual is defined as a collection of fragments used to generate a ligand. Each fragment within this collection is a gene and is represented by its unique index within the source table of the library fragments. A rank weighting is calculated and assigned for each index within the source (parent) ligands. These are calculated by the index of the fragments within the input table, and a rank weighting function is described below
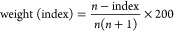
where *n* is the length of the table (number of fragments). The weight index provides larger fractional weights to higher ranked fragments toward the top of the list, which are expected to produce ligands of a tighter binding affinity because they were generated from ligands with tighter binding affinity than others. These ranked weights are used to define a categorical distribution using the random.choices() function, which is used to select indexes when new fragments are being incorporated.

**Figure 2:**
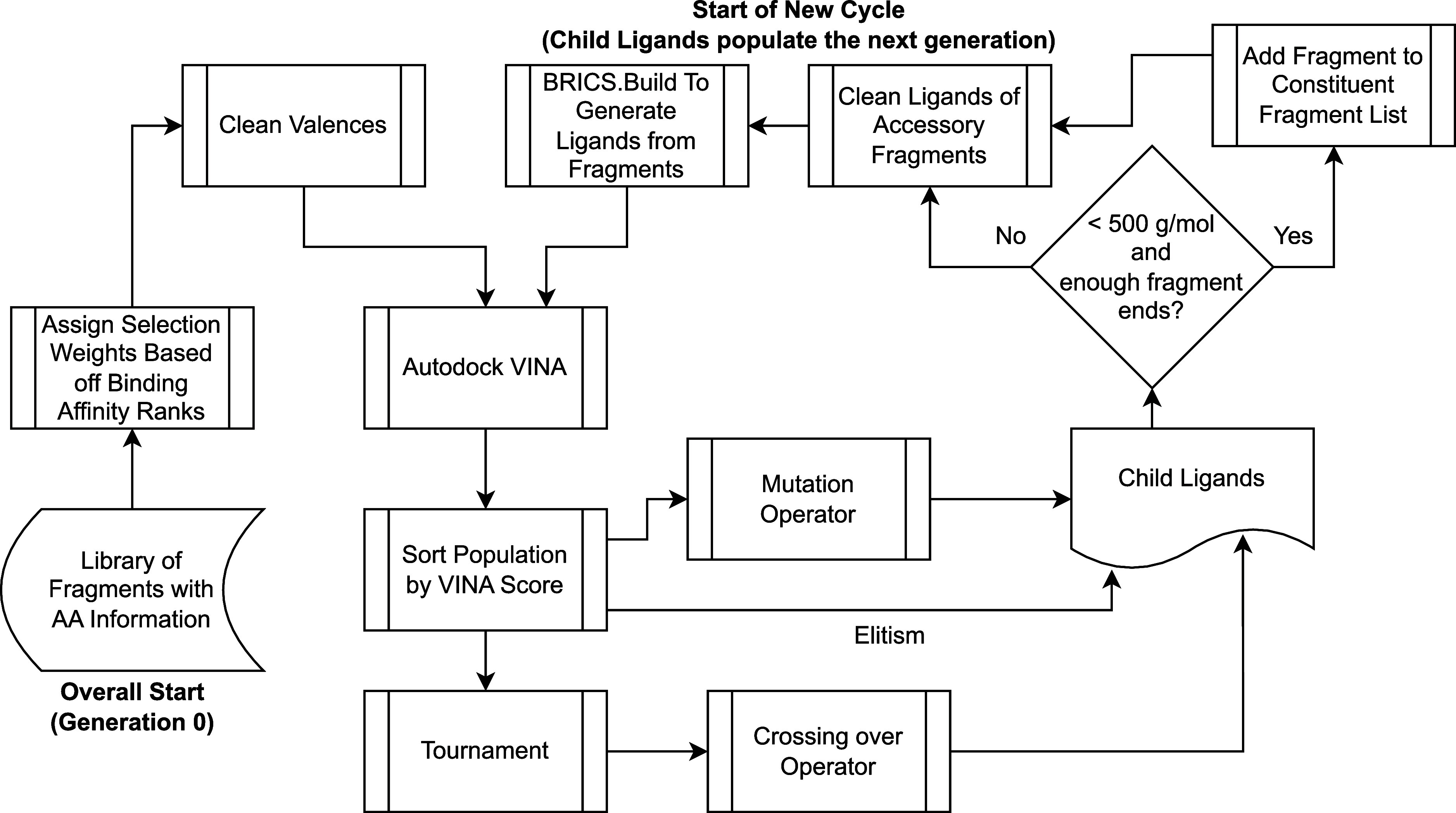
In the first phase of ligand optimization, a genetic algorithm is used to create ligands from the fragments produced by the prescreening and fragmentation pipeline shown in Figure 1 from Wilson et al.^68^ Fragments are represented like genes and assigned a weighted rank to determine the selection probability. Initially, fragments are randomly chosen and evaluated using Autodock VINA (see “Autodock Vina” block) and optionally analyzed for drug likeness via QED scores. Subsequent ligand generations are crafted using mutation (“Mutation” block), crossover, and elitism strategies, abiding by specific molecular weight and fragment use rules. The ligands are further cleaned to ensure that all fragments are utilized in the resultant ligand (“clean ligands” block) and constructed into new generations using the BRICS.BUILD module in RDKit (“BRICS.Build generates ligands” block).

Figure 2 shows an overview of the genetic algorithm procedure. To start, a random selection of fragments is chosen to seed the first generation. Hydrogens are added to unfilled valences (see the clean valences block in Figure 2), and they are run through Autodock VINA to evaluate them (see the Autodock Vina block in Figure 2). In addition, quantitative effectiveness of drug-likeness (QED) scores can be optionally calculated to determine drug likeness, and a combined QED and VINA score can be used for evaluating candidate ligands in the population instead of the VINA score alone in the procedures described below.

The next generation is determined on the basis of three operators: mutation, crossing over, and elitism. To start, the population is sorted by VINA score (the sort population by VINA score block in Figure 2), and the top 5/8th of the population are chosen to be run through the mutation operator (the mutation block in Figure 2). These ligands, whether mutated or not, are added to the next generation. Next, a tournament selection takes place (the tournament block in Figure 2), where each individual is compared against two other randomly chosen individuals, and the individual with the lowest binding affinity is chosen to be a parent used in the crossing over operator. Two unique individuals that win a tournament are run through the operator at a time, and the operator returns two children, which will both be included in the next generation. The crossing over operator accounts for 1/4th of the following generation. The last 1/8th of the next generation is developed using elitism, where the individuals with the lowest binding affinity in the parent population are added without alteration.

The children to be used in the next generation are screened to determine whether they have hit their maximum molecular weight of 500 g/mol. If not, or the number of fragment ends (unfilled valences) is an odd number, an additional fragment selected based the on off-the-rank weighting function described above is added to the ligand (the add fragment to constituent fragment list block in Figure 2). If the maximum weight has been reached or the number of fragment ends is even, the ligands remain unaltered and are added to the fragment constituent list and progress to the clean ligands phase of the cycle.

To ensure that every fragment included in an individual is incorporated into the resultant ligand, each ligand is run through a cleaning function (the clean ligands of accessory fragments block in Figure 2) to ensure that there are enough fragment ends to accommodate all fragments. The BRICS.BUILD module in RDKit, which is used to generate each ligand from its constituent fragments, will not accommodate a fragment if there is not an end to which it will bind to. Therefore, fragments which exceed the number of ends available to attach fragments are pruned to avoid including them as a gene in future generations when they did not contribute to the evaluated structure. After running through the cleaning function, the BRICS.BUILD module generates ligands from the fragments (see BRICS.Build generates ligands from fragments in Figure 2), and the children replace the parents as the new population. Then, the next generation begins.

### 2.4 Genetic Algorithm Components

#### 2.4.1 Mutation Operator

Based on the mutation rate supplied by the user, each fragment has a chance of mutating. In the event of a mutation, another fragment is substituted for the existing fragment, which is selected based off the categorical distribution calculated at the beginning using the random.choices() function. The ligands, mutated or not, are incorporated into the next generation.

#### 2.4.2 Crossing Over Operator

The crossing over operator requires two parents to be input as well as a user-supplied crossing over rate. If a crossing over occurs, then a random index is selected between both individuals, and the fragments (indexes) between both individuals are swapped after that point. For instance, assume an instance in which two individuals with four corresponding fragments each are selected as parents:

Parent 1: [314, 132, 4813, 192]. Parent 2: [102, 8512, 591, 5123].

The indexes correspond to fragments in the original source CSV. If a crossing over event occurs, a random index is chosen as the index to perform the switch. Suppose index 2 is selected. The following child ligands will be generated:

Child 1: [314, 132, 591, 5123]. Child 2: [102, 8512, 4831, 192].

These individuals will be incorporated into the next generation.

#### 2.4.3 Generating Ligands from Fragments

The build function from the BRICS package of RDKIT is used to generate ligands. The function is set up to output only complete SMILES, negating the need to manually add hydrogens to unfilled valences.

### 2.5 Iterative Fragment Addition (Phase 2 of Fragment Synthesis)

The second stage of optimization is iterative fragment addition, which is effectively a hill-climbing algorithm for maximizing the drug design objective. In this study, we considered both binding affinities alone, as predicted by AutoDock VINA, and binding affinity in combination with the QED score. The QED score was developed by Bickerton et al.^82^ as an improvement over rules, such as Lipinski’s Rule of Five,^83^ which combines different thresholds and properties of ligands that tend to be associated with successful drugs. The QED score is calculated by a formula based on a weighted sum of “desirability functions”, i.e., molecular properties associated with desirability for a particular class of drugs. In this paper, we use the default definition of QED from Bickerton et al., i.e., including molecular weight (MW), octanol–water partition coefficient (ALOGP)^24^, number of hydrogen bond donors (HBD), number of hydrogen bond acceptors (HBA), molecular polar surface area (PSA), number of rotatable bonds (ROTB), the number of aromatic rings (AROM), and number of structural alerts (ALERTS).^82^ The QED score is computed using RDKit’s qed() module (https://www.rdkit.org/docs/source/rdkit.Chem.QED.html). The algorithm proceeds the same in either the QED + binding affinity or affinity-only, except that for QED + binding affinity, the optimization criterion is a sum of the two scores.

The iterative fragment addition methodology requires a list of premade starter ligands and a data set including fragments associated with amino acids of the target protein. The list of premade starter ligands is the output of the genetic algorithm, while the data set of ligands and associated amino acids is the output of the initial prescreening and fragmentation pipeline. Before running the fragment addition, the fragment data set is converted to a dictionary, where each amino acid is a key with a maximum of 100 associated fragments based on the median binding affinity of parent ligands.

**Figure 3:**
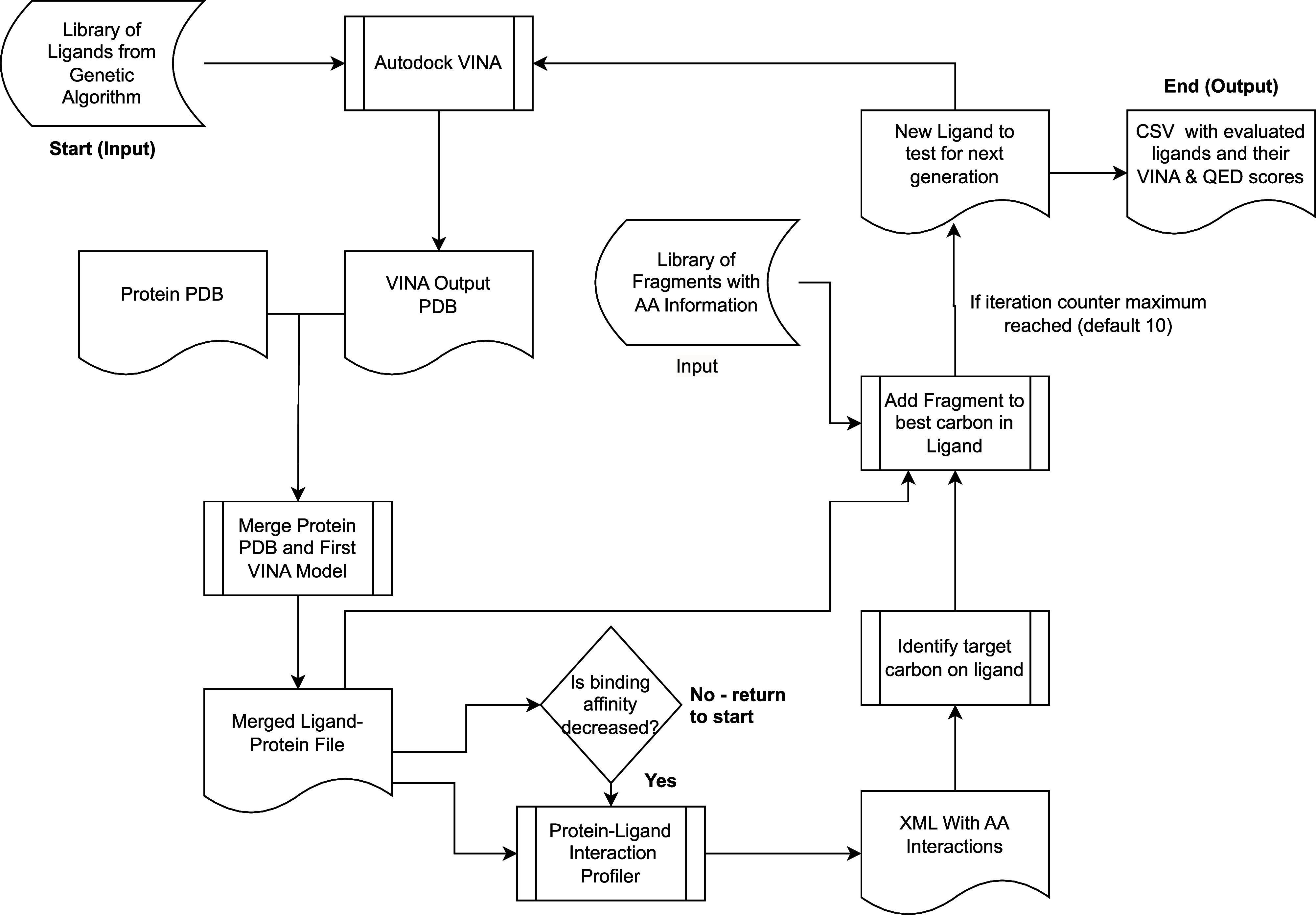
Iterative fragment addition stage, shown schematically here, can begin with any kind of starting ligand but in our method begins with candidate ligands synthesized through the previous genetic algorithm-based ligand synthesis phase (see Figure 2) and fragments obtained from the initial ligand prescreening and fragmentation pipeline. The optimization objective of this phase is the binding affinity score predicted by AutoDock VINA, alone or in a sum with the quantitative effectiveness of drug-likeness (QED) score, which evaluates the beneficial molecular properties for drug design. The methodology begins with premade starter ligands and an amino acid-associated fragment data set. Through successive iterations, each ligand is evaluated and possibly merged with a protein PDB file for further assessment by protein–ligand interaction profiler (PLIP). Fragments are strategically added to target regions of the ligand, ensuring optimal binding affinity and maintaining molecular weights under 700 g/mol to ensure viable drug targets. This process cyclically refines ligand structures, using tools such as RDKit for optimization and 3D structuring, continuing to a prescribed iteration limit or until an optimizable ligand is generated.

Figure 3 shows an overview of the iterative fragment addition stage. At the start of each iteration, each ligand in the population is evaluated using Autodock VINA, using the same grid box and seeding as described above for the ligand prescreening stage. Next, the first model in the PDB output file of Autodock VINA is merged with the receptor protein’s PDB file. This step is in preparation for the evaluation of the space using protein–ligand interaction profiler (PLIP). Before running PLIP, each ligand in the population is compared against its predecessor to determine if the fragment addition was successful at decreasing binding affinity. If binding affinity decreased, then the ligand is ready for another attempt at addition. If binding affinity increased, then the addition was unsuccessful at decreasing binding affinity, and the predecessor replaced the current ligand before the addition of a new fragment.

Next, all of the merged ligand–protein PDB files with molecular weights less than 700 g/mol are run through PLIP. Ligands with molecular weights greater than 700 g/mol are deemed too large for addition, as larger ligands take increasingly longer to evaluate using VINA and tend to make for worse drug targets. PLIP outputs an XML file containing information about the relevant amino acids in the binding pocket of the protein. It also includes the distances of the ligand to each of these amino acids. These distances are compared against distances between ligand carbons and protein carbons in the merged ligand–protein PDB, and the closest ligand carbon to an amino acid is selected as the target region to add a fragment.

After the target carbon is identified, the ligand and fragment are merged into one MOL object. A bond is formed between the target carbon in the ligand and the atom bound to a dummy atom (indicating a fragment end). All dummy atoms in the fragment are converted to hydrogens to fill the valence of the ligand. The new molecule is embedded into a 3D structure and is MMFF94-optimized. In the event that RDKit is unable to optimize the newly generated ligand, the next best target carbon is selected, and another fragment addition is attempted. This is repeated multiple times until an optimizable ligand is generated or the number of iteration attempts reaches ten. If the iteration counter limit is reached, the loop ends, and the SMILES of every ligand and associated VINA and QED scores are written to a CSV file. If the iteration counter limit is not reached, the new ligands are evaluated using Autodock VINA, and the cycle repeats.

Occasionally, RDKit will be able to embed and optimize the combined ligand and fragment MOL object but will be unable to embed and optimize the SMILES generated from the MOL object. For this reason, a filter is included for every generation that tests to make sure that each ligand can be converted from its SMILES to an optimized 3D ligand. SMILES that cannot be converted will not be included in the final CSV file. This prevents the inclusion of invalid ligands in the final output, which cannot be converted to 3D MOL objects.

## 3 Results

### 3.1 Assessing Performance of Genetic and Iterative Optimization Ligand Designs Based on Prescreening Information

To determine the effect of fragment quality on the iterative algorithm results, four pools of fragments are created to be fed into the iterative algorithm for each protein target. Each pool is collected from the same prescreening data set sourced from the prescreening and fragmentation pipeline. The worst pool (WP) runs use the worst 1000 fragments by ligand binding affinity. The large pool (LP) runs use all fragments regardless of binding affinity. The unprioritized (U) and prioritized (P) runs use the same data set of fragments, where up to 1000 of the best fragments per unique amino acid are included in the pool. The distinction between the two runs is that the P runs pair fragments with interacting amino acids sourced from PLIP. This difference tests the effect of matching fragments with associated amino acids rather than randomly assigning fragments. The WP, LP, and U runs all add random fragments within the pool, while the P runs target fragments toward specific amino acids.

**Figure 4:**
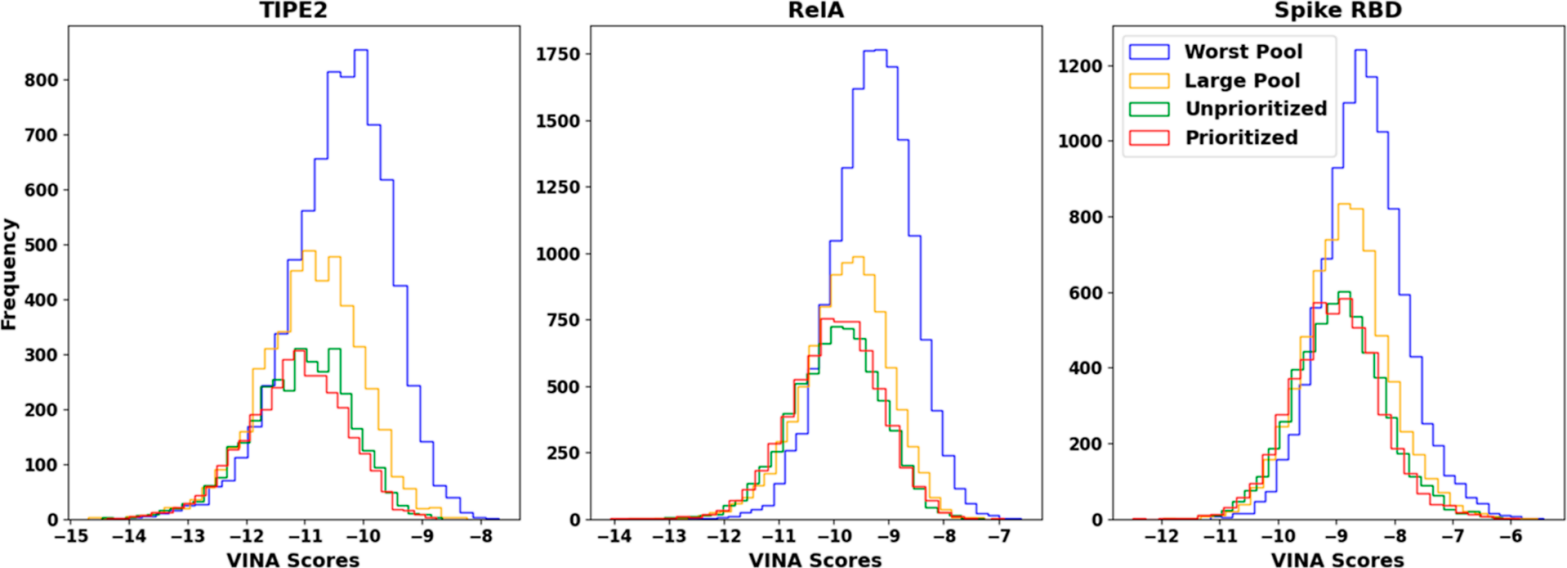
Histograms comparing fragment pool generation methodologies. The top three graphs included comprehensive results of each iterative run. The “worst pool” trial used the worst 1000 fragments by VINA scores from the source fragment data set. The “large pool” included all fragments. The “unprioritized” and “prioritized” trials used the same subset of fragments generated with priority of the VINA score and the top 1000 fragments associated with a given amino acid. Unprioritized trials use randomly assigned fragments in the pool to bind to the ligands, while prioritized trials use subpools for each amino acid. For a given target amino acid, the prioritized trial suggested a fragment known to have interacted with that amino acid in the past based off the PLIP screening.

The histograms in Figure 4 highlight the final run results for each protein target. The maximum ligand size producible by the algorithm is 700 g/mol. Percentile scores and top median pools are highlighted because they tend to be from which leads are chosen . The percentile scores describe how good the distribution of ligands is toward the top of the results, while the top median scores describe how improved the very best ligands are.

The P and U plots are shifted left relative to LP and WP runs, indicating a bias toward producing ligands with better binding affinities. For all protein targets, the median and mean VINA scores improve in order of WP, LP, U, and P. In addition, the 95th, 97th, and 99th percentile scores highlight a significant decrease in VINA scores at the top end of each data set, with significant improvements in VINA scores in the U and P runs relative to the LP and WP runs. However, the U and P runs tend to have percentile scores within ±0.01 kcal/mol of each other, indicating negligible differences in scores between one another. The top 10 to 50 median scores for RelA and Spike RBD show a similar pattern, where median scores improve from WP to LP to U/P. Interestingly, the ligands generated for the TIPE2 target did not show a similar trend in scores, with LP, U, and P top 10 to 50 median scores demonstrating no clear trend between runs. The top 10 median LP run even outperformed the U and P runs at −14.21 kcal/mol compared to −14.04 and −13.98 kcal/mol, respectively. Outlier ligands are often produced by chance. Addition of a fragment to a given carbon may on rare occasions significantly improve binding affinity over targeted efforts to improve the overall distribution of ligands. Therefore, ligands toward the top of the results do not follow the trend of improving binding affinities in the order of WP, LP, and U/P.

Although the LP and WP runs have lower median binding affinities, they overall produce significantly more ligands relative to the P and U trials, as per the count column of each run in Table 1 for each of the protein targets. This is expected as fragments toward the top of the fragment data set tend to have larger molecular weights. Each addition in the U and P runs increases the molecular weight higher than an addition in the WP and LP runs, which causes the U and P runs to reach the max molecular weight of 700 g/mol more rapidly. This causes the U and P runs to produce fewer unique ligands than the LP and WP runs.

**Table 1:** Iterative Run Statistics

run	worst pool	large pool	unprioritized	prioritized
(a) TIPE2 (in kcal/mol)				
median	–10.34	–10.86	–11.04	–11.12
mean	–10.41	–10.91	–11.09	–11.15
mode	–10.19	–10.59	–10.53	–11.3
count	7480	4605	3318	3188
95th percentile	–11.89	–12.37	–12.58	–12.59
97th percentile	–12.18	–12.61	–12.85	–12.84
99th percentile	–12.78	–13.22	–13.38	–13.33
top 50 median	–13.3	–13.43	–13.48	–13.46
top 20 median	–13.58	–13.82	–13.84	–13.78
top 10 median	–13.86	–14.2	–14.04	–13.98
best ligand	–14.45	–14.69	–14.46	–14.37
(b) RelA (in kcal/mol)				
median	–9.28	–9.75	–9.92	–9.94
mean	–9.31	–9.79	–9.96	–9.97
mode	–10.05	–10.11	–10.01	–10.19
count	15,328	9218	6710	6356
95th percentile	–10.53	–11.13	–11.34	–11.33
97th percentile	–10.72	–11.38	–11.54	–11.55
99th percentile	–11.05	–11.83	–11.94	–12.02
top 50 median	–11.67	–12.26	–12.38	–12.45
top 20 median	–11.98	–12.5	–12.78	–12.84
top 10 median	–12.28	–12.72	–13.0	–13.2
best ligand	–12.74	–13.27	–13.74	–14.06
(c) Spike RBD (in kcal/mol)				
median	–8.54	–8.84	–8.95	–9.01
mean	–8.53	–8.83	–8.95	–9.0
mode	–8.525	–10.21	–10.08	–10.02
count	10,148	6629	5362	4845
95th percentile	–9.66	–10.03	–10.19	–10.23
97th percentile	–9.84	–10.21	–10.41	–10.41
99th percentile	–10.17	–10.52	–10.74	–10.78
top 50 median	–10.59	–10.77	–10.98	–10.98
top 20 median	–10.81	–10.98	–11.3	–11.24
top 10 median	–11.0	–11.14	–11.48	–11.46
best ligand	–11.17	–11.97	–11.99	–12.49

The ligands showing the best binding affinity for TIPE2 were analyzed for potential synthetic paths, and the synthesis schemes are shown in the Supporting Information. Other candidate ligands that have strong binding affinity and favorable solubility and other drug-likeness properties are also shown in Supporting Information, Figure S3. Overall, the best ligands for RelA (−14.06 kcal/mol) and TIPE2 (−14.69 kcal/mol) have greater binding affinity and SpikeRBD (−12.49 kcal/mol) has approximately equal binding affinity as compared to the highest binding affinity for ligands generated using a simple greedy search in our previous work describing the prescreening and fragment generation pipeline.^68^ Unlike the proposed method described in this paper, the previous method simply added the fragments with the highest median parent binding affinity, resulting from prescreening without any heuristic or iterative optimization.

### 3.2 Comparison of the Proposed Methodology with Other Genetic and Deep Learning Fragment-Based Methods

In addition to the experiments described above, trials of other ligand optimization packages are shown here to compare the effectiveness of the described state-of-art genetic and iterative (machine learning) methodologies. One exemplary package that takes a genetic approach is AutoGrow4. Due to limitations in ligand pool size, for the trials shown here, AutoGrow4 is fed a random sample of 1000 source ligands from the top 10,000 ligands used by the fragmentation pipeline and was run 10 times. All default variables and packages were used, and the file conversion package selected is obabel. Each run is allowed to run for 30 generations, at which time scores between generations tend to plateau. The same receptors and search boxes used by the genetic and iterative codes are supplied to AutoGrow4.

**Figure 5:**
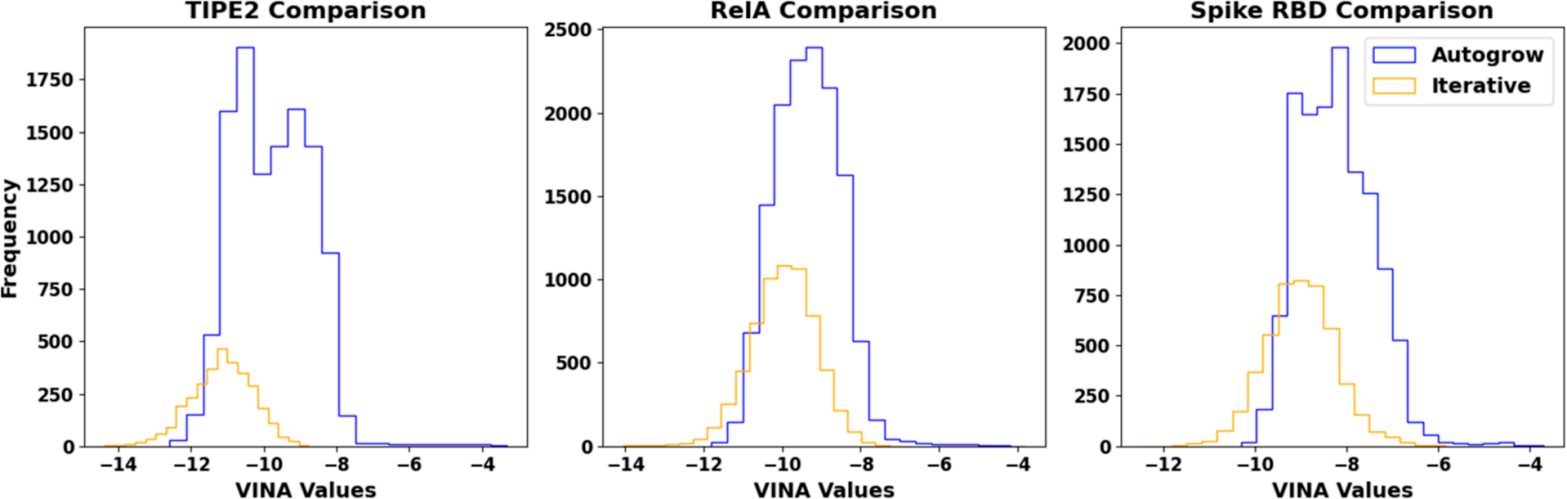
Comparison of AutoGrow4 and FDSL-DD iterative generated ligands. The proposed method’s histogram data was selected from prioritized trials described in Section 1.

The histograms in Figure 5 highlight significantly higher binding affinities for ligands generated by AutoGrow4 relative to binding affinities generated by the iterative methodology. This is further highlighted in the tables, which show significant improvements in binding affinity in the percentile score columns and top ligand median score columns. Interestingly, AutoGrow4 appeared to generate a better overall median score in the RelA trial. The best ligands produced by AutoGrow4 in all 10 runs for each target protein had VINA scores of −12.6, −11.8, and −10.3 kcal/mol for TIPE2, RelA, and Spike RBD respectively, while the best iterative ligands from the prioritized trials were −14.37, −14.06, and −12.49 kcal/mol.

The second-stage iterative optimization of the proposed approach appears to produce significantly fewer ligands than AutoGrow4, as indicated by the counts in Table 2. This is attributable to the iterative approach only being able to add fragments onto ligands, which means it will hit the molecular weight ceiling faster than an exclusively genetic approach like AutoGrow4. AutoGrow4 can mix and match substructures within a given population of molecules, allowing for more combinations and, hence, more unique ligands.

**Table 2:** AutoGrow4 Comparison (in kcal/mol)

	TIPE2		RelA		Spike RBD	
run	AutoGrow4	prioritized	AutoGrow4	prioritized	AutoGrow4	prioritized
median	–9.8	–11.12	–9.4	–9.94	–8.3	–9.01
mean	–9.77	–11.15	–9.41	–9.97	–8.25	–9.0
mode	–10.7	–11.3	–9.1	–10.19	–8.5	–10.02
count	11,142	3188	13,760	6356	12,215	4845
95th percentile	–11.3	–12.59	–10.7	–11.33	–9.4	–10.23
97th percentile	–11.5	–12.84	–10.8	–11.55	–9.5	–10.41
99th percentile	–11.8	–13.33	–11.1	–12.02	–9.7	–10.78
top 50 median	–12.2	–13.46	–11.4	–12.45	–9.9	–10.98
top 20 median	–12.4	–13.78	–11.6	–12.84	–10.0	–11.24
top 10 median	–12.45	–13.98	–11.7	–13.2	–10.1	–11.46
best ligand	–12.6	–14.37	–11.8	–14.06	–10.3	–12.49

**Figure 6:**
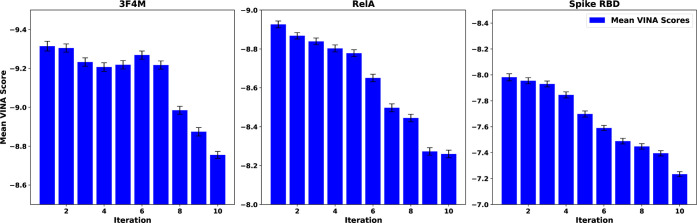
Mean VINA scores of each iteration in the DeepFrag runs using default DeepFrag fragments (i.e., from the model training data) are plotted along with the unbiased standard error of the mean for each iteration as error bars. Scores tend to increase per iteration of DeepFrag, indicating worsened binding affinities. The best VINA scores for each protein target are −12.17, −11.47, and −9.895 kcal/mol for TIPE2, RelA, and Spike RBD, respectively. The graph is plotted such that the *y*-axis values decrease from bottom to top to show stronger binding affinities as higher values. The *y*-axis range differs for each target to illustrate the similarity in trend between the targets differently for each target.

The proposed approach is also compared to DeepFrag, a deep learning approach that aims to predict the best fragment to add to a ligand within a binding pocket. The default fragments in the DeepFrag library are used in this comparison, and 10 runs are completed for each target. To create a fair comparison, for each iteration, the best ligand proposed by DeepFrag is used as the input ligand for the next iteration. Ten iterations are completed per run. The intermediate ligands produced by DeepFrag are combined into one data set which is sorted by the DeepFrag scoring function. Due to computational constraints, a sample of the best 10,000 ligands from this combined data set is run through Autodock VINA. A histogram comparison would be ineffective at comparing the entire population of ligands generated by the proposed methodology to a sample of the DeepFrag results, so a bar plot is used to represent the results in Figure 6.

The trend in the bar plots in Figure 6 shows that DeepFrag appears to produce poor ligands for the target binding pockets. Even when comparing best scores, DeepFrag produces significantly worse scores than the best scores of the proposed iterative methodology, at −14.37, −14.06, and −12.49 kcal/mol for TIPE2, RelA, and Spike RBD, respectively. Interestingly, the average scores tend to trend down, indicating that DeepFrag may not be optimized to improve the binding affinity generated by Autodock VINA. This may be caused by DeepFrag being overfitted to the training set used, limiting the extension to other protein targets like the ones presented in this study. Because the model is not tuned for the tested protein targets, the ligands generated drift into higher Autodock VINA scores, indicating decreased (poorer) binding affinities.

Figure 6 shows the results of using the fragments on which the DeepFrag model was trained, rather than the fragments obtained as a result of the FDSL-DD prescreening pipeline.^73^ To determine whether using fragments from the FDSL-DD source ligands used in this paper would perform better, we used DeepFrag’s open source software to feed them as input fingerprints, as shown in the Supporting Information. Supporting Information, Figure S1 illustrates that DeepFrag generally performed less well on fragments from the source ligands, with binding affinities deteriorating much faster through each iteration than the runs using DeepFrag default fragment library shown in Figure 6. This is to be expected, given that the DeepFrag model was fit to the fragments in the default library and targets used in training, rather than the specific drug targets analyzed in this paper. We also analyzed the diversity of DeepFrag- and Autogrow-synthesized ligands as compared to the source ligand libraries and ligands synthesized using FDSL-DD and two-stage optimization as described here. As Supporting Information, Table 1 shows, DeepFrag generates ligands that are more diverse than the source ligands but notably with much lower binding affinity; therefore, it does not seem like DeepFrag is able to prioritize which fragments from the source library are helpful for ligand synthesis. On the other hand, AutoGrow was found to be much more similar to the source ligands, perhaps at the cost of not improving binding affinity significantly, as shown in Figure 5. Despite the Autogrow-synthesized ligands being more similar to the source ligands, they were not estimated to be more synthetically accessible, as calculated by the method described in ref (84), than the proposed method or DeepFrag-synthesized ligands, as shown in Supporting Information, Figure S2.

### 3.3 Evaluating Multi-objective Optimization for Drug-Likeness and Binding Affinity Based on Prescreening Information

To show that the proposed method can also account for drug likeness properties in addition to binding affinity and still produce viable candidate ligands, a straightforward modification can be made to render the optimization multiobjective. Specifically, in addition to evaluating binding affinity using Autodock VINA, the genetic and iterative algorithms contain an optional multiobjective scoring function. The multiobjective scoring function considers the QED score, a single metric generated from drug desirability functions, in addition to VINA binding scores. This scoring function can be customized depending on a user’s optimization interests. The algorithm combines a ligand’s VINA and QED *z*-scores into a single value, giving equal weight to both. The *z*-scores are calculated relative to the original ligand data set, from which the fragments were sourced. This methodology ensures that marginal gains in VINA performance do not significantly reduce drug likeness.

**Figure 7:**
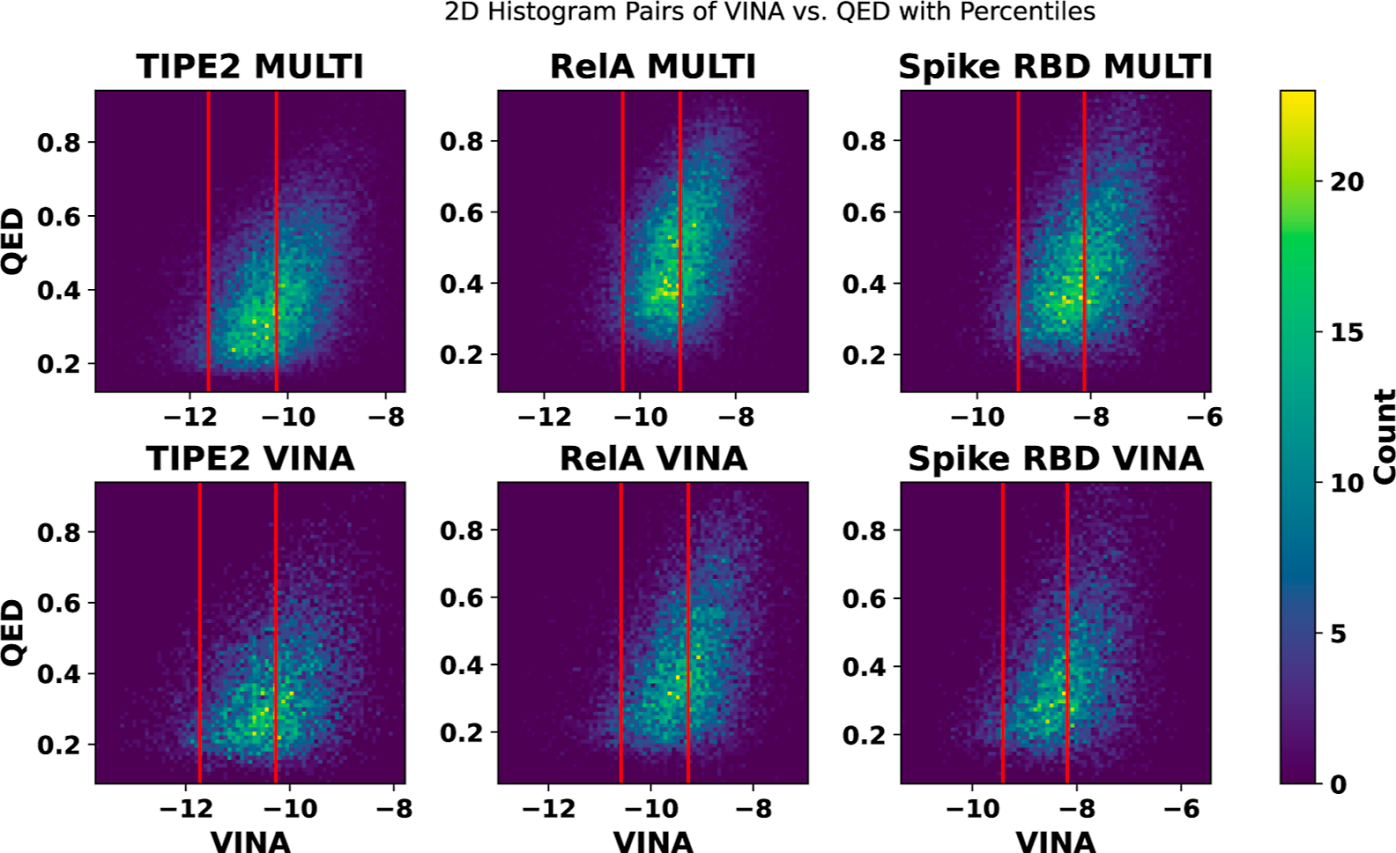
Histograms comparing multiobjective and VINA prioritizations over final VINA and QED scores using iterative approach. Lower VINA scores indicate improved binding affinity, and higher QED scores indicate better drug-likeness. Red lines indicate 50th and 95th percentile scores, which are selected to segment the regions of each data set. Both iterative runs are on the same set of starting ligands from the genetic algorithm, including multiobjective prioritization. The starting ligand set is selected based on best multiobjective scores.

The graphs in Figure 7 compare the generated ligands using the multiobjective approach compared to a VINA prioritization approach. Both approaches are run on the same set of starter ligands generated from the genetic algorithm, which is run using the multiobjective evaluation function. Table 3 summarizes the statistics for each run for the respective protein targets.

**Table 3:** Multiobjective Iterative Run Statistics (in Kcal/mol)

	TIPE2		RelA		spike RBD	
run	MULTI	VINA	MULTI	VINA	MULTI	VINA
median	–10.23	–10.27	–9.16	–9.27	–8.12	–8.18
mean	–10.24	–10.29	–9.17	–9.3	–8.12	–8.19
mode	–10.22	–10.19	–10.01	–10.07	–8.177	–8.398
count	24748	7784	24083	11886	19440	9041
95th percentile	–11.63	–11.73	–10.36	–10.58	–9.28	–9.42
97th percentile	–11.83	–11.94	–10.55	–10.77	–9.43	–9.62
99th percentile	–12.25	–12.31	–10.9	–11.17	–9.71	–9.91
top 50 median	–13.0	–12.73	–11.57	–11.73	–10.2	–10.28
top 20 median	–13.27	–12.99	–11.87	–12.03	–10.39	–10.42
top 10 median	–13.5	–13.16	–12.0	–12.29	–10.62	–10.56
best ligand	–13.93	–13.74	–12.96	–12.98	–11.34	–11.37

**Figure 8:**
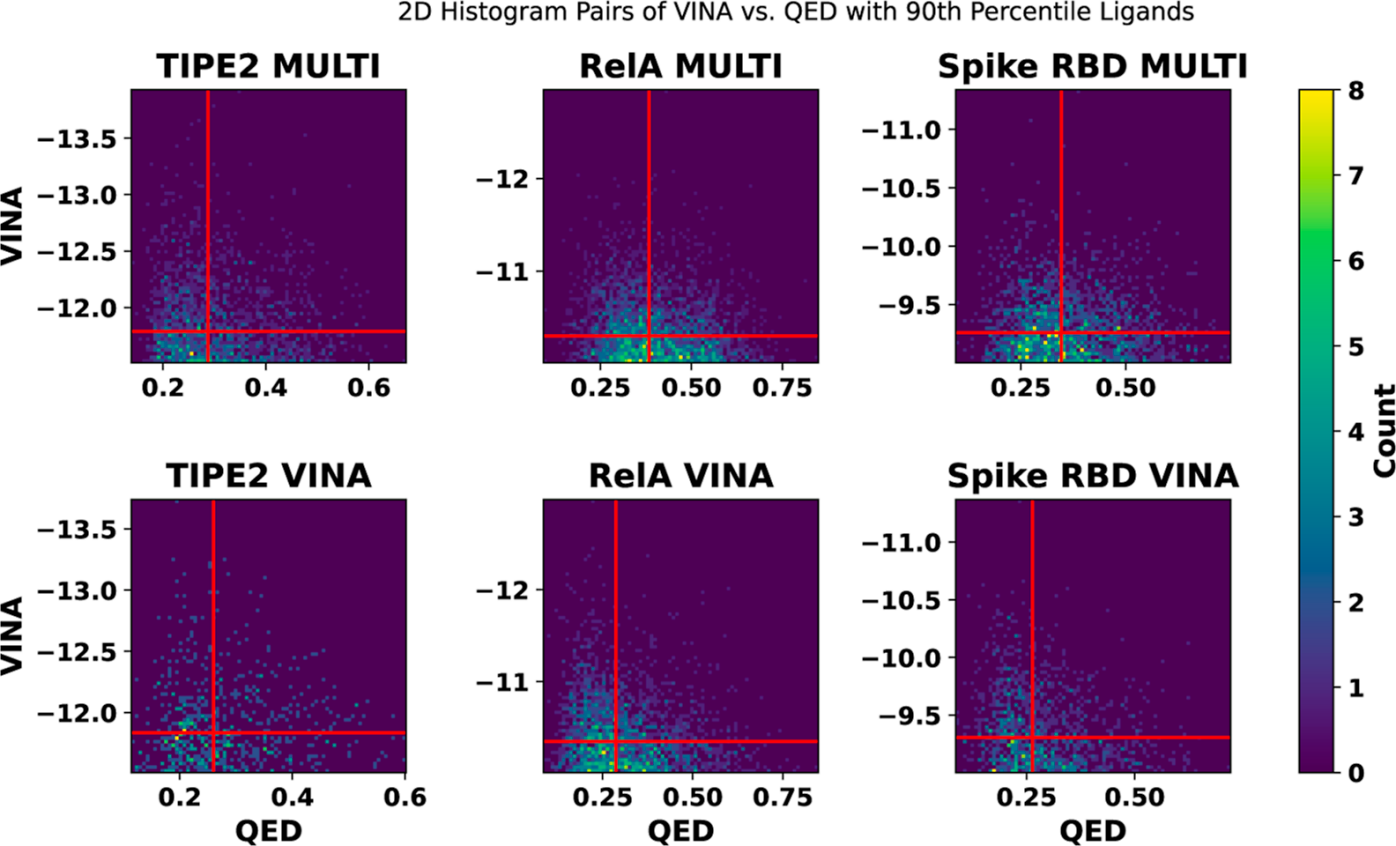
Focusing on the strongest ligand candidates obtained from multiobjective optimization, the histograms shown here plot the 95th percentile ligands by VINA score with QED score for each of the protein targets. The horizontal line indicates the 97.5th percentile VINA score. The vertical line indicates the 50th percentile QED score of the 95th percentile ligands by the VINA score.

The plots in Figure 7 demonstrate that the multiobjective runs produce similar VINA score distributions to the VINA prioritization runs. Although the multiobjective prioritization produces slightly worse percentile scores, it produced better top 50, 20, and 10 median scores during the TIPE2 runs and a better top 10 median score during the spike RBD run, as shown in Table 3. Figure 8 indicates that the multiobjective function produces ligands with similar binding affinities to the VINA prioritization while significantly improving QED scores, even at the top of the data sets where VINA scores tend to be improved but QED scores tend to be lower.

Similar to the large pool and worst ool trials described above, the multiobjective runs produced far more ligands than the VINA prioritization runs, for example, as indicated by the counts in Table 3. However, both trials used the same fragment pools. The reason for this difference is that the multiobjective trials account for drug desirability, which tends to prefer smaller ligands. If a given iteration produces a marginal gain in the VINA score but a large gain in molecular weight, the algorithm will backtrack as the multiobjective score will not have improved, even if the VINA score did. Therefore, more unique ligands are produced as each iteration needs to improve the VINA score significantly enough to outweigh any decreases in the QED score.

Interestingly, the ligands generated by multiobjective optimization also tend to be more similar to the source ligands (i.e., input to prescreening) when evaluated using a measure of distance that accounts for both fragment structure similarity and chemical properties, as described in Supporting Information, Material and shown in Supporting Information, Table 1. Moreover, the ligands generated by multiobjective optimization are predicted to be more synthetically accessible, using the estimate of synthetic accessibility described in ref (84), as shown in Supporting Information, Figure 3 and Table 2.

We further studied the three-dimensional conformations of ligands generated by iterative optimization (following the evolutionary stage) within the receptor binding pocket with particular attention to analyzing the molecular fragments within them and comparing those to fragments obtained from the source ligand databases. The findings are detailed in the Supporting Information, Figures S4–S6 and the accompanying text. We particularly consider two issues with the proposed method: (1) the extent to which the fragment-based approach described here is in fact advantageously benefiting by incorporating fragments from the original ligand library, i.e., the degree to which the orientation of the fragments is substantially altered in the process of optimally assembling synthetic ligands and (2) potential misalignment in the synthesized ligands’ binding orientations and determining whether the optimization process results in ligands that can effectively form bonds within binding pockets in the targets in a manner similar to the source ligands.

In summary, the results demonstrate that the most favorable synthetic ligands generated through the proposed optimization method indeed likely have potentially realistic structures and ligand–receptor bonds. Even after fragments are reconstituted into the novel ligands, they generally retain or assume conformations conducive to effective binding, as in source ligands from which the fragments were originally derived through the first stage of the FDSL-DD pipeline (albeit providing lower binding affinity in the context of those source ligands). The analysis in the Supporting Information thereby further illustrates that the two-stage optimization strategy retains at least some capacity to maintain the integrity of fragment interactions within the target protein’s binding pocket. Moreover, the interactions show a strong binding profile between the computationally synthesized ligand and key residues in the protein receptor binding pockets.

## 4 Discussion

As the results presented in this paper illustrate, the fragments from the ligands drug discovery (FDSL) pipeline can significantly improve computational ligand design and optimization by prioritizing fragments based on the results of initial virtual screening. By prioritizing fragments with higher potentials for success, the FDSL-DD pipeline not only increases the efficiency of the subsequent drug design process but also leads toward yielding compounds with optimal binding affinities and drug-like properties. Moreover, the FDSL-DD pipeline includes ligand-binding domain analysis such that key carbons for binding are identified and prioritized during the iterative step, thereby improving optimization as well. The two-stage optimization method described in this paper further shows more favorable binding affinities and more diverse results than simply picking the top of the list from the FDSL-DD pipeline, which was the approach used in our previous work.^68^

Specifically, the results show that finely tuned fragment pools, including fragments involving ligands with more favorable binding affinities, produce larger shares of ligands with good binding affinities relative to pools without prioritization. Additionally, the similar performance of the prioritized trials (fragment pools associated with amino acids) and unprioritized trials (prioritized fragment pools without amino acid association) hints at a minimal influence of specifying fragments to specific amino acids during the fragment addition process. Outlier ligands with significant performance often emerge due to chance, which blurs the observable trends at the top tier of each resulting data set. This can be attributed to fragments within the large pool (pool with all fragments) and worst pool (pool with worst 1000 fragments by binding affinity) that significantly improve binding affinity in contexts not apparent in the source ligand data set. Furthermore, fragments included in the prioritized and unprioritized pools with higher associated binding affinities tend to be heavier, hitting the maximum molecular weight of 700 g/mol in fewer iterations and thus producing fewer unique ligands than in the large pool and worst pool runs. This upper limit is selected to avoid limiting max ligand sizes to 500 g/mol according to Lipinski’s Rule of Five, which, as others have pointed out, are likely to be outdated and, if used too strictly, may detrimentally filter out otherwise promising leads.^85^ Raising the upper limit beyond 700 g/mol may result in ligands displaying stronger binding affinities. These ligands would be better suited for the large binding pockets of the target proteins under consideration. However, it is important to note that this limit is chosen to enhance computational efficiency. Using larger ligands in Autodock VINA significantly increases the computational time required, which may limit the number of unique ligands screened within a given time frame.

The proposed method is tested on three distinct types of targets, each with its own unique challenges. First, we tested protein targets associated with solid cancer tumorigenesis, with the aim of better cancer treatments. Our second design target relates to the issue of antimicrobial resistance, a significant concern, as rising resistance could render basic infections untreatable. Third, we applied the proposed method to the spike protein receptor binding domain (RBD) of COVID-19, where inhibiting this domain might prevent the virus from entering human cells, offering a potential therapeutic avenue. The robustness of this approach is further reinforced by its capacity to handle the varied geometries, binding conditions, and chemical conditions presented by these targets. Navigating different geometries means understanding diverse target structures while adapting to unique binding conditions and varying chemical environments showcases the method’s versatility.

The methodology yields ligands with enhanced binding affinities when compared to those of other methods. This improvement may be due to the capability of the genetic and iterative algorithms adapted for the FDSL-DD pipeline in this paper to accommodate larger source ligand data sets. In contrast, AutoGrow4 and DeepFrag have limitations in terms of source ligand data set size. The scalability of the proposed methodology enables a more extensive exploration and analysis of the chemical space within the binding pocket prior to ligand generation. Scalability, in turn, makes it possible for the genetic and iterative algorithms to include even more information for constructing new ligands and fine-tuning them toward improved binding affinities than that demonstrated in this paper. It is also important to highlight that a crucial advantage of using genetic optimization over the deep learning approach in DeepFrag is that it does not require time-consuming and computationally costly (generally requiring multiple GPUs) training to generate ligands. Indeed, DeepFrag using fragments from the source database performs even worse than DeepFrag using default fragments on which it was originally trained. For DeepFrag to be more successful, it would require retraining the model on the specific targets analyzed here, which would be much slower than the two-stage optimization method shown here.

Balancing optimal binding affinity with favorable drug-likeness properties is essential for successful lead identification in drug discovery. The method as currently developed employs QED scores. The results demonstrate that employing a multiobjective evaluation can produce candidate leads that can have superior drug-likeness properties, such as solubility, with strong binding affinity. In particular, multiobjective prioritization produces a more diverse pool of ligands compared to VINA prioritization. The generated ligands demonstrate significant improvements in QED scores, with minimal losses in binding affinity. Moreover, by prioritizing QED scores, modest improvements in binding affinity do not override significant decreases in drug-likeness, resulting in ligands with both improved binding affinities and drug-likeness.

Notably, some of the candidate leads with strong binding affinity obtained using the proposed method, e.g., as shown in Supporting Information, Figure 3, have ESOL scores between −4 and −6, indicating moderate solubility.^86^ To prepare for in vitro studies, polar groups may be manually added to further improve solubility, although these changes may affect the predicted binding affinity. Other computational estimations of solubility, such as *M* Log *P*, can also be assessed to determine if log *P* scores are less than 5, which is in agreement with Lipinski’s Rule of Fives.^83^ Moreover, there are plausible synthetic pathways for candidates with the strongest binding affinity, as shown in the Supporting Information for exemplary candidates for TIPE2.

Although the prioritized trials did not yield significant improvements in binding affinity relative to the unprioritized trials, further pool screening should be completed in future studies to determine whether amino acid matching can yield stronger ligands. For instance, matching fragment properties to amino acid properties instead of exclusively relying on PLIP analysis may yield more optimal fragment-amino acid pairings, improving the ligand binding affinity upon addition. Future studies may also consider adding other objectives for optimization.

Finally, the proposed method relies on computationally predicted rather than actual experimental data on the initial ligand population while still providing useful information to guide the drug design and optimization process. Not only is the prescreening data generated in silico, thereby avoiding costs and complexity of in vitro screening, but the prescreening is also done using relatively low computational cost and scalable computer docking methods, as opposed to the more costly and less scalable molecular dynamics methods. Accordingly, the FDSL and optimization methods presented in this paper are highly scalable. To further scalability, the code developed to implement both the genetic and iterative optimization stages herein is fully parallelized and can be readily executed across multiple processors in a computing environment as the ligand population and fragment pool increases.

## Data Availability

The source code for the optimization methods described in this paper has been made publicly available for noncommercial use only at https://github.com/EESI/FDSL_Evo. The scripts used for running the trials shown in this paper are also available at Github, and they can provide a guide for implementation in other high-performance computing environments. The files used as inputs to the optimization pipeline and raw results of the optimized pipeline shown in this paper are also provided at the same Github site.

## Supporting Information Available

The Supporting Information is available free of charge at https://pubs.acs.org/doi/10.1021/acs.jcim.4c00234. For raw result and input data files, please see the Github page for this paper at https://github.com/EESI/FDSL_Evo

Additional DeepFrag experiments; difference (diversity) of generated ligands relative to source ligands; estimated synthetic accessibility; identifying structures for potential candidate ligands; proposed synthetic pathway for candidate ligands; structural analysis of computationally synthesized ligands (PDF)
