# Supplementary material for: Streamlining Computational Fragment-Based Drug Discovery through Evolutionary Optimization Informed by Ligand-Based Virtual Prescreening
Source: J Chem Inf Model. 2024 May 2;64(9):3826–40. doi: 10.1021/acs.jcim.4c00234 (PMC11197033; doi:10.1021/acs.jcim.4c00234)
Supplement: Supplementary file 1 — ci4c00234_si_001.pdf [file ci4c00234_si_001.pdf]

Supplementary Material for *Streamlining Computational Fragment-Based Drug Discovery through Evolutionary Optimization Informed by Ligand-Based Virtual Prescreening*, Rohan Chandraghatgi, Hai-Feng Ji, Gail L. Rosen, and Bahrad A. Sokhansanj

Table of Contents

I. Additional DeepFrag Experiments *2*

II. Difference (Diversity) of Generated Ligands Relative to Source Ligands *2*

III. Estimated Synthetic Accessibility *4*

IV. Identifying Structures for Potential Candidate Ligands *8*

V. Proposed Synthetic Pathway for Candidate Ligands *10*

VI. Structural Analysis of Computationally Synthesized Ligands *11*

1. **TIPE2** **12**
2. **RelA** **16**
3. **Spike RBD** **20**

I. ADDITIONAL DEEPFRAG EXPERIMENTS

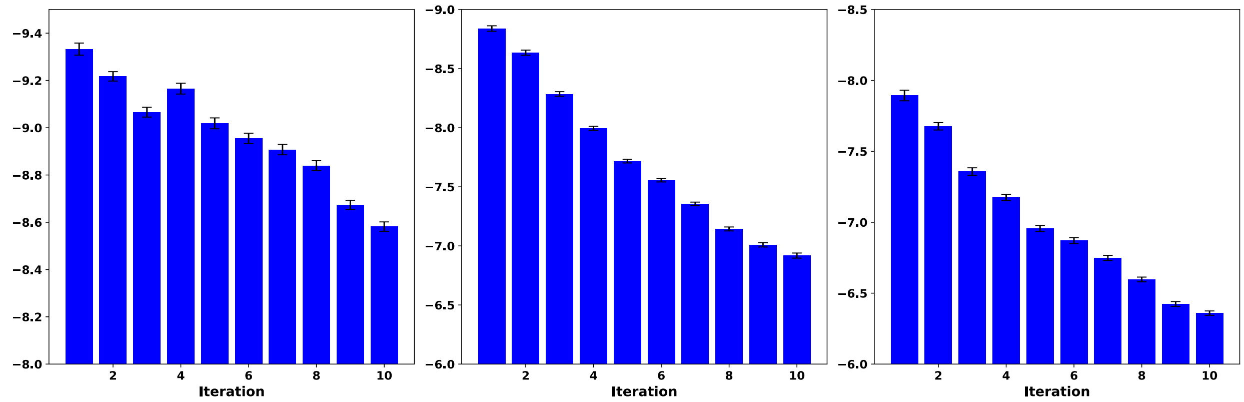

Supplementary Figure 1. The mean VINA scores of each iteration in the DeepFrag runs are plotted along with the unbiased standard error of the mean for each iteration as error bars. Scores tend to increase per iteration of DeepFrag, indicating worsened binding affinities. The fragments provided for testing DeepFrag are the fragments generated from the prescreening step for each of the three targets. The fragments, which were generated by BRICS, had to be modified to use in DeepFrag by hydrogenating fragmented ends. Subsequently, the Python scripts made available at the Durrant laboratory's DeepFrag Github site[1] are used to generate a molecular fingerprint file for the fragments saved in HDF5 format as required by DeepFrag. As the plots show here, the scores tend to increase per iteration of DeepFrag, indicating worsened binding affinities. The graph is plotted such that the y-axis values decrease from bottom to top to show stronger binding affinities as higher values. The y-axis range differs for each target to illustrate the similarity in trend between the targets differently for each target. Generally, for all three targets, the ligands that are generated have worse binding affinities in each iteration as compared to the default fragments used for DeepFrag's training data (compare to Fig. 6).

II. DIFFERENCE (DIVERSITY) OF GENERATED LIGANDS RELATIVE TO SOURCE LIGANDS

We compare the difference, or diversity between the candidate ligands generated using the proposed method and the original source ligands used to generate the fragment library. The metric used for diversity is Frechet Chemnet Diversity (FCD), described in Kristina Preuer, Philipp Renz, Thomas Unterthiner, Sepp Hochreiter, and Günter Klambauer, Fréchet ChemNet Distance: A Metric for Generative Models for Molecules in Drug Discovery, *Journal of Chemical Information and Modeling*, 2018, 58 (9), 1736-1741. DOI: 10.1021/acs.jcim.8b00234. FCD leverages the ChemNet neural network and the mathematical concept of the Fréchet distance between curves, which has previously been applied to evaluate generative methods in

[1] *See* https://github.com/durrantlab/deepfrag/blob/main/scripts/make_fingerprints.py.

computer vision. The FCD method calculates the Fréchet distance between distributions of molecules in the ChemNet feature space, thereby accounting for both molecular structure and functional properties. The FCD does have the limitation that it can only be meaningfully determined when the number of molecules in the distributions is identical; accordingly, in the calculations shown here the distributions are downsampled to 1000 randomly selected compounds.

As shown in Supplementary Table 1, [2] the two-stage optimization method described in this paper generates greater diversity from source ligands than AutoGrow, but is generally closer than DeepFrag even when using the original fragments. As expected, DeepFrag with the default fragment library is the most distinct, which is to be expected as the they do not come from the source ligands and fragment structure is one of the bases for FCD. (The minimal FCD of "Prescreening" represents a baseline that is not exactly 1, i.e., minimum distance, because the FCD score is based on a sampling of the populations.) Notably, the results of multiobjective optimization are chemically more similar to the source ligands, suggesting that they have the most desirable drug-like properties.

Supplementary Table 1. Computed Fréchet ChemNet Distance (FCD) score between a random sample of 1000 source ligands (i.e. input to prescreening) and a random sample of the populations as set forth below.

Target	Dataset	FCD to Source
TIPE2	Prescreening	1.9649
TIPE2	Multiobjective	20.629
TIPE2	Prioritization	24.1407
TIPE2	Large Pool	22.2774
TIPE2	Worst Pool	20.4859
TIPE2	Autogrow	13.7107
TIPE2	DeepFrag (Default)	25.0851
TIPE2	DeepFrag (Prescreen)	23.5004
Spike RBD	Prescreening	1.9343
Spike RBD	Multiobjective	18.8321

[2] The computations in Supplementary Table 2 are made using the FCD module available at https://github.com/bioinf-jku/FCD. As indicated in the documentation, RDKit Smiles canonicalization is used. Subsamples of 1000 randomly selected compounds are used on a NVidia T4 GPU running in the Google Colab environment.

Target	Dataset	FCD to Source
Spike RBD	Prioritization	21.484
Spike RBD	Large Pool	20.1535
Spike RBD	Worst Pool	20.0927
Spike RBD	Autogrow	15.5939
Spike RBD	DeepFrag (Default)	29.2805
Spike RBD	Deep Frag (Prescreen)	25.4281
RelA	Prescreening	2.0226
RelA	Multiobjective	14.9333
RelA	Prioritization	18.4124
RelA	Large Pool	17.6747
RelA	Worst Pool	16.6252
RelA	Autogrow	15.2094
RelA	DeepFrag (Default)	23.9287
RelA	DeepFrag (Prescreen)	26.0315

III. ESTIMATED SYNTHETIC ACCESSIBILITY

The synthetic accessibility of the generated ligands is estimated by calculating a synthetic accessibility (SA) score using the method described in Ertl, P., Schuffenhauer, A. Estimation of synthetic accessibility score of drug-like molecules based on molecular complexity and fragment contributions. *J Cheminform* **1**, 8 (2009). https://doi.org/10.1186/1758-2946-1-8. The score is determined based on features that are common among synthesized compounds identified in the PubChem database by fragmenting representative PubChem compounds and identifying common fragmentized features. That score is then corrected by substracting a “complexity penalty,” which includes a ring complexity component (i.e. based on the numbers of ring bridge and spiro atoms. a stereocomplexity score (counting number of stereo centers), the number of macrocycles, and a size penalty based on the molecular weight. Supplementary Fig. 2 shows the synthetic accessibility scores for the ligands in pools identified in the paper text. The scores are computed using the sascorer module, which is a contribution to the rdkit module, with code obtained from Github.[3] Supplementary Table 2 shows the medians of the synthetic accessibility (SA) score.

[3] *See* https://github.com/rdkit/rdkit/blob/master/Contrib/SA_Score/sascorer.py

Supplementary Figure 2. Predicted synthetic accessibility scores for pools described in the Results section of the paper. These include the results of the iterative (second optimization step) for the Prioritization, Large, and Worst pools based on prescreening, ligands generated based on multiobjective optimization (i.e. binding affinity and solubility), and ligands generated by the Autogrow and DeepFrag (default fragment library) methods. Notably, while the ligands generated by the proposed method are, as expected, higher (less favorable) in scoring they do have overlap with the source ligands, which consistent ligands that have been synthesized. Overall, the generated ligands are well within the range of predicted synthesizable compounds, i.e. with scores in the 3 to 4 range, and well less than 6 which Ertl and Schuffenhauer suggested represent compounds that are particularly difficult to synthesize.

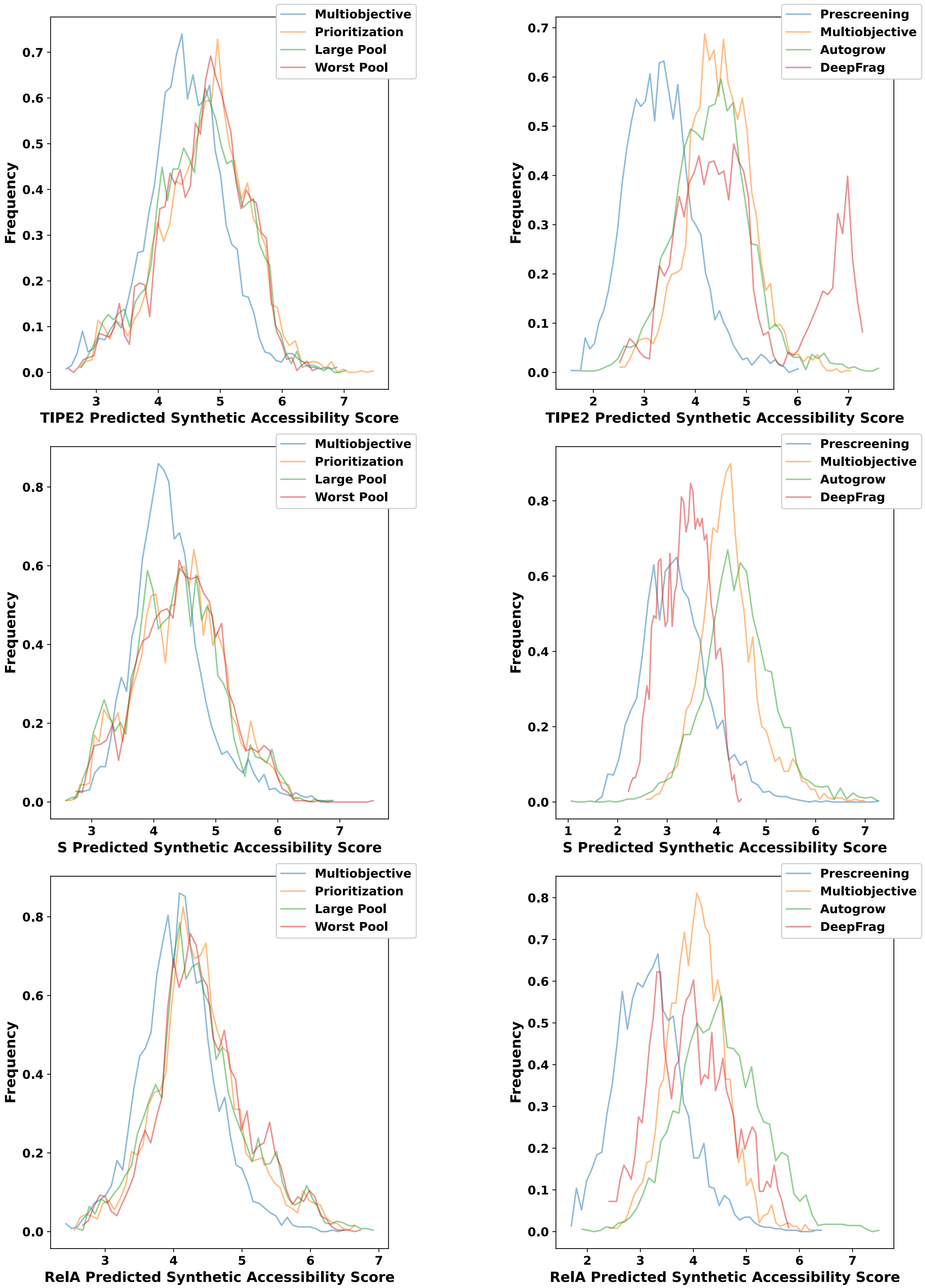
Multiobjective
Prioritization
Large Pool
Worst Pool
Frequency
TIPE2 Predicted Synthetic Accessibility Score
Prescreening
Multiobjective
Autogrow
DeepFrag
Frequency
TIPE2 Predicted Synthetic Accessibility Score
Multiobjective
Prioritization
Large Pool
Worst Pool
Frequency
S Predicted Synthetic Accessibility Score
Prescreening
Multiobjective
Autogrow
DeepFrag
Frequency
S Predicted Synthetic Accessibility Score
Multiobjective
Prioritization
Large Pool
Worst Pool
Frequency
RelA Predicted Synthetic Accessibility Score
Prescreening
Multiobjective
Autogrow
DeepFrag
Frequency
RelA Predicted Synthetic Accessibility Score

Supplementary Table 2. Median estimated synthetic accessibility (SA) score for datasets (described in the Results section) for the three targets.

Target	Dataset	Median SA Score
TIPE2	Prescreening	3.2656
TIPE2	Multiobjective	4.4427
TIPE2	Prioritization	4.8196
TIPE2	Large Pool	4.7322
TIPE2	Worst Pool	4.7971
TIPE2	Autogrow	4.3371
TIPE2	DeepFrag	4.5532
Spike RBD	Prescreening	3.1517
Spike RBD	Multiobjective	4.1861
Spike RBD	Prioritization	4.4502
Spike RBD	Large Pool	4.4039
Spike RBD	Worst Pool	4.4766
Spike RBD	Autogrow	4.4046
Spike RBD	DeepFrag	3.3887
RelA	Prescreening	3.1516
RelA	Multiobjective	4.0613
RelA	Prioritization	4.3347
RelA	Large Pool	4.3016
RelA	Worst Pool	4.3504
RelA	Autogrow	4.465
RelA	DeepFrag	3.9356

IV. IDENTIFYING STRUCTURES FOR POTENTIAL CANDIDATE LIGANDS

To illustrate the kinds of structures that are produced by the proposed pipeline and optimization methods, the 2D structure, and SMILES strings of three potential candidate ligands for each of the targets analyzed in this paper (TIPE2, RelA, and Spike RBD) are shown in Supplementary Fig. 3. These ligands, for example, may be evaluated in future \textit{in vitro} studies for binding affinity and druggability. The candidate structures shown here are selected from the results of both the default-objective and multi-objective functions described in previous sections. The criteria used to select potential exemplary candidates to display in Table Supplementary Fig. 3 are (i) to minimize binding affinity as predicted by Autodock VINA (specifically targeting predicted affinities of less than -13 kcal/mol or as close as possible where targets were not found in that range), (ii) estimated solubility (ESOL) scores indicating moderate solubility or better calculated to be between -4 and -6 using the method described by Delaney,[4] and (iii) molecular weights of less than 700 g/mol. Additional quantitative values for drug-likeness properties are predicted by SwissADME[5] and shown Supplementary Fig. 3. The SMILES strings for the structures in Supplementary Fig. 3 are reproduced below:

**Target**	**Label**	**SMILES**
TIPE2	(i)	CC[C@@H]1Cc2ccccc2N(c2c(O)ccc3ccc(N4c5ccccc5CCc5ccccc54)cc23)c2ccccc21
TIPE2	(ii)	c1ccc2ccc(N3c4ccccc4CCc4ccccc43)cc2c1N1c2[C@@H](N2CCCCC2)c2ccccc21
TIPE2	(iii)	Oc1ccc2ccc(N3c4ccccc4CCc4ccccc43)cc2c1N1c2ccccc2C[C@@H](C2CCCCC2)c2ccccc21
RelA	(iv)	O=C1[C@H]2[C@H]3CC[C@H](C3)[C@H]2C(=O)N1c1ccc2nnc(N3C(=O)[C@H]4[C@@H]5C[C@H]NC6=CC7=c8ccccc8=C[C@@H]7C=C6)[C@@H](C5)[C@H]4C3=O)n2n1
RelA	(v)	O=C1[C@H]2[C@@H]3CC[C@@H]([C@H]3N3CCN4CCCC[C@@H]4C3)[C@H]2C(=O)N1c1ccc2nnc(N3C(=O)[C@H]4[C@@H]5CC[C@@H](C5)[C@H]4C3=O)n2n1
RelA	(vi)	CC1NC(=O)N(N2CCc3ccc(-c4c(-c5ccc6c(c5)C(=O)NCC6)oc5ccccc5c4=O)cc3C2=O)C1=O
Spike	(vii)	C[C@]1(c2ccc3c(=O)n4c(nc3c2)[C@H]([C@@]2(C)NC(=O)NC2=O)CCCCC4)NC(=O)N([C@@H]2NN=C
Spike	(viii)	O=C1N=C(c2nc(N3C(=O)c4ccccc4N4[C@@H](c5noc(-c6cccc(=O)[nH]6)n5)NN[C@@H]34)no2)CC=C1N1NC(=O)[C@@H]2CC=CC[C@H]2C1=O
Spike	(ix)	O=c1nc(-c2cnn3c2[nH]c(=O)c2ccccc23)n2c3c(c(-c4cnn5c4[nH]c(=O)c4ccccc45)ccc13)CCC2

[4] Delaney, J. S. ESOL: Estimating Aqueous Solubility Directly from Molecular Structure. *J Chem Inf Comput Sci* **2004**, *44* (3), 1000–1005. https://doi.org/10.1021/ci034243x.

[5] Daina, A.; Michielin, O.; Zoete, V. SwissADME: A Free Web Tool to Evaluate Pharmacokinetics, Drug-Likeness and Medicinal Chemistry Friendliness of Small Molecules. *Sci Rep* **2017**, *7* (1), 42717. https://doi.org/10.1038/srep42717.

Supplementary Figure 3. Molecular structures of potential candidate ligands Generated by the FDSL and two-stage optimization pipeline.

(a) Candidate Ligands for TIPE2

(i)

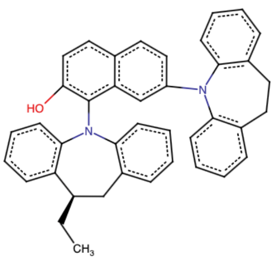

CC[C@@H]1Cc2ccccc2N(c2c(O)ccc3ccc(N4c5ccccc5CCc5ccccc54)cc23)c2ccccc21

(ii)

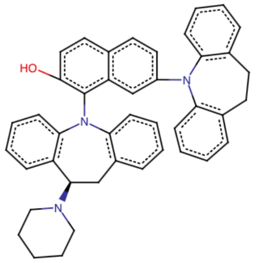

c1ccc2ccc(N3c4ccccc4CCc4ccccc43)cc2c1N1c2ccccc2C[C@@H](N2CCCCC2)c2ccccc21

(iii)

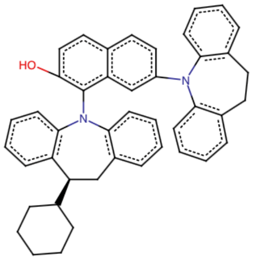

Oc1ccc2ccc(N3c4ccccc4CCc4ccccc43)cc2c1N1c2ccccc2C[C@@H](C2CCCCC2)c2ccccc21

(b) Candidate Ligands for RelA

(iv)

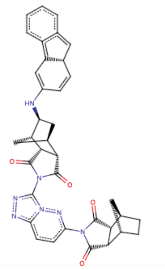

O=C1[C@H]2[C@H]3CC[C@H](C3)[C@H]2C(=O)N1c1ccc2nnc(N3C(=O)[C@H]4[C@@H]5C[C@H](NC6=CC7=c8ccccc8=C[C@@H]7C=C6)[C@@H](C5)[C@H]4C3=O)n2n1

(v)

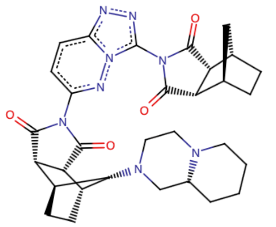

O=C1[C@H]2[C@@H]3CC[C@@H]([C@H]3N3CCN4CCCC[C@@H]4C3)[C@H]2C(=O)N1c1ccc2nnc(N3C(=O)[C@H]4[C@@H]5CC[C@@H](C5)[C@H]4C3=O)n2n1

(vi)

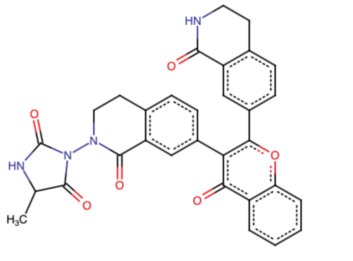

CC1NC(=O)N(N2CCc3cc(-c4c(-c5ccc6c(c5)C(=O)NCC6)oc5ccccc5c4=O)cc3C2=O)C1=O

(c) Candidate Ligands for Spike RBD

(vii)

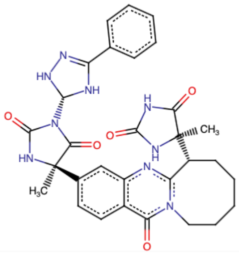

C[C@]1(c2ccc3c(=O)n4c(nc3c2)[C@H]([C@@]2(C)NC(=O)NC2= O)CCCCC4)NC(=O)N([C@@H]2NN=C(c3ccccc3)N2)C1=O

(viii)

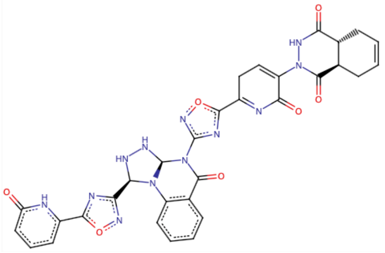

O=C1N=C(c2nc(N3C(=O)c4ccccc4N4[C@@H](c5noc(-c6cccc(=O)[nH]6)n5)NN[C@@H]34)no2)CC=C1N1NC(=O)[C@@H]2CC=CC[C@H]2C1=O

(ix)

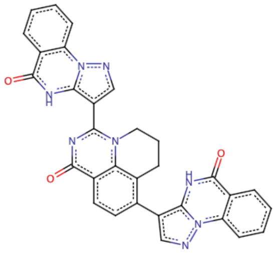

O=c1nc(-c2cnn3c2[nH]c(=O)c2ccccc23)n2c3c(c(-c4cnn5c4[nH]c(=O)c4ccccc45)ccc13)CCC2

(d) Quantitative Data for the Candidate Ligands

	Binding Affinity (kcal/mol)	MW (g/mol)	ESOL	MLogP	TPSA (Å²)	Log Kp (cm/s)	GI	BBB
(i)	-14.59	558.73	-10.46	7.51	26.71	-2.08	Low	No
(ii)	-14.27	613.81	-10.50	7.16	29.95	-2.70	Low	No
(iii)	-13.98	612.82	-11.79	8.18	26.71	-1.23	Low	No
(iv)	-12.83	625.68	-5.60	4.59	129.87	-7.97	High	No
(v)	-12.29	584.67	-4.30	3.41	124.32	-8.87	High	No
(vi)	-12.18	548.55	-5.41	2.51	129.03	-7.42	High	No
(vii)	-11.40	597.62	-4.61	2.69	178.92	-8.75	Low	No
(viii)	-11.24	676.60	-4.47	0.43	237.15	-9.93	Low	No
(ix)	-11.12	552.54	-5.65	4.94	135.21	-7.55	Low	No

V. PROPOSED SYNTHETIC PATHWAY FOR CANDIDATE LIGANDS

To illustrate the synthesizability of candidate ligands generated by this method, we show here proposed synthetic pathways for candidate ligands with the best binding affinities for selected targets. In particular the best candidates for TIPE2 are analyzed below, with predicted AutoDock VINA binding affinities of -14.6, -14.3, and -14.0 kcal/mol respectively, and they are shown below along with SMILES strings (see also the top row of Supplementary Fig. 3):

(i)

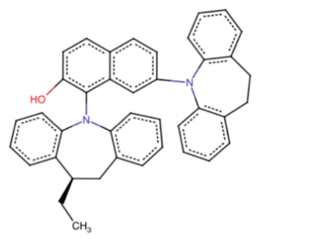

CC[C@@H]1Cc2ccccc2N(c2c(O)ccc3ccc(N4c5ccccc5CCc5ccccc54)cc23)c2ccccc21

(ii)

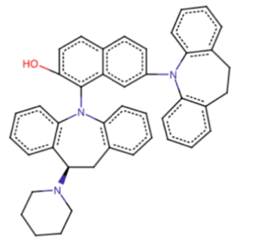

c1ccc2ccc(N3c4ccccc4CCc4ccccc43)cc2c1N1c2ccccc2C[C@@H](N2CCCCC2)c2ccccc21

(iii)

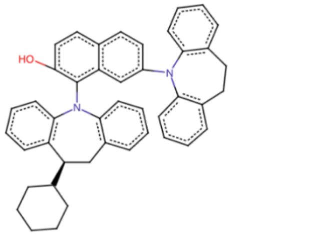

Oc1ccc2ccc(N3c4ccccc4CCc4ccccc43)cc2c1N1c2ccccc2C[C@@H](C2CCCCC2)c2ccccc21

These top compounds are chosen for evaluating synthetic feasibility and their binding affinity (KI) and biochemical efficacy (IC_{50}). For instance, synthesis of compounds 1-3 will be completed following the synthetic Scheme 1-3, respectively. Based on their biophysical and enzymatic inhibition results, they will be further finetuned so the compounds can pass the Lipinski's rule of five and other rules for druggability. The finetuned analogs with various modifications will be synthesized using a similar method with various starting materials.

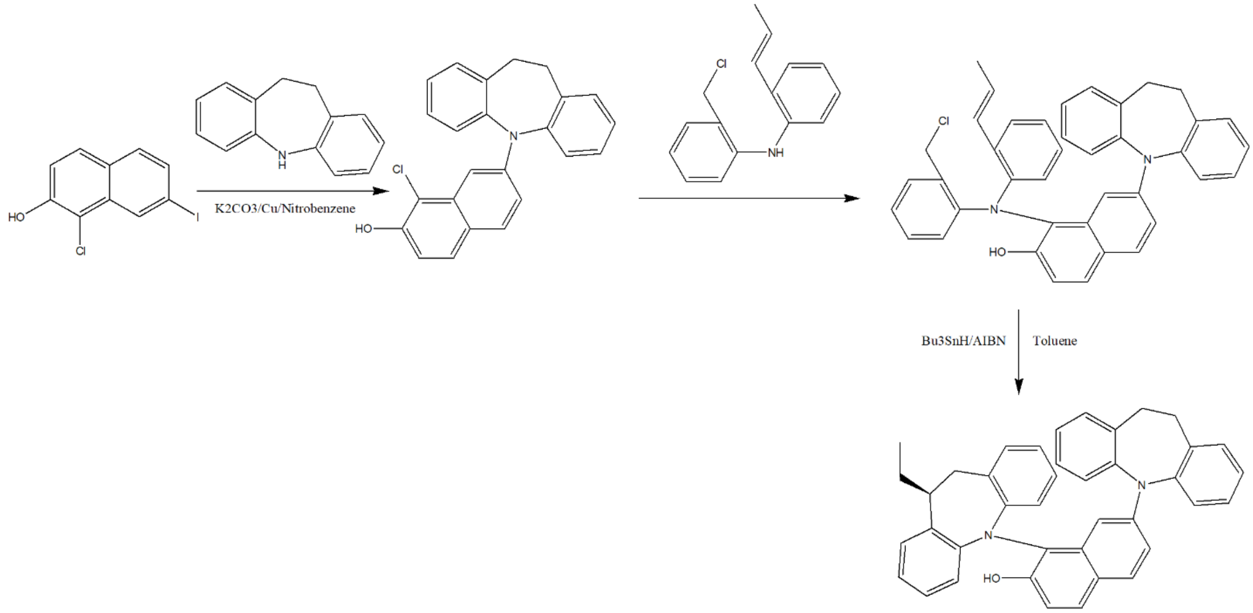

Scheme 1. Synthesis Route of compounds 1

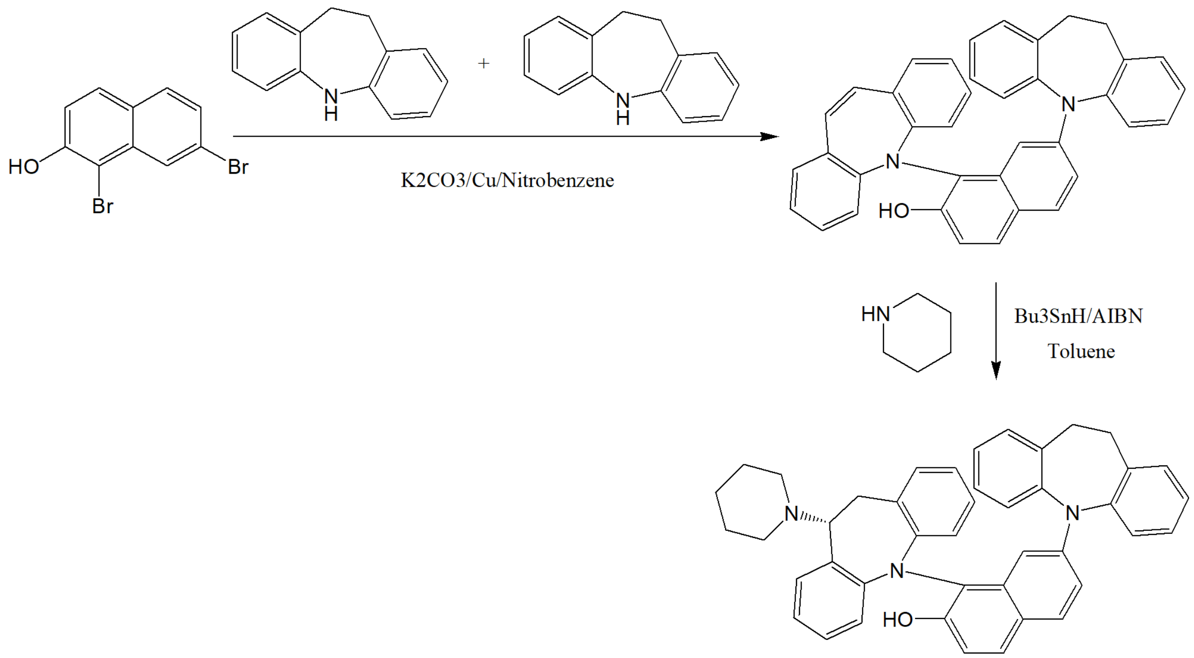

Scheme 2. Synthesis Route of compounds 2

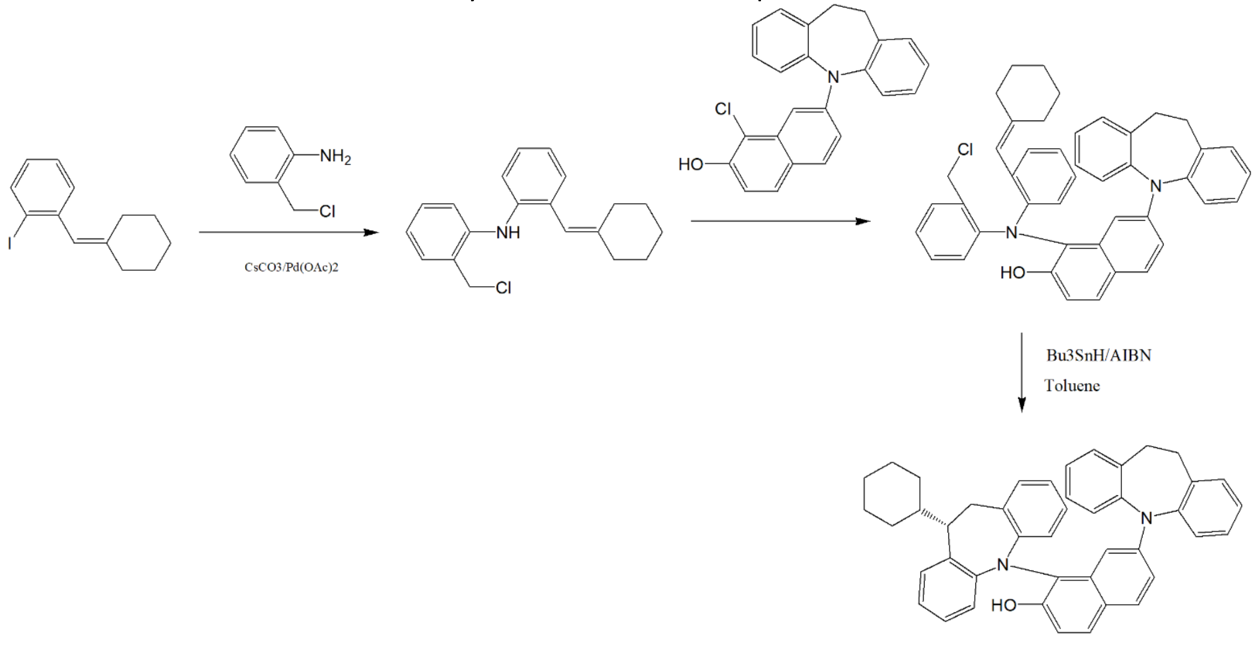

Scheme 3. Synthesis Route of compounds 3

VI. STRUCTURAL ANALYSIS OF COMPUTATIONALLY SYNTHESIZED LIGANDS

We further perform a structural analysis of the bonding between the computationally synthesized ligands with receptor binding pockets, as well as specifically analyzing the three-dimensional conformations to confirm how source fragments might change conformation as they proceed through the genetic and iterative optimization stages of the proposed

methodology. Fragment conformation might change through the course of the optimization process. Notably, the MMFF94Optimize function may change the conformation of the fragment within the generated ligand following synthesis, eliminating much of the potential benefit of retaining the 3D conformation of the fragments, i.e., as they were pre-fragmentation.

To analyze the binding of computationally synthesized ligands with receptor pockets, we utilize Autodock VINA for ligand screening, followed by a combination of structural merging with the Protein-Ligand Interaction Profiler (PLIP) to predict bonds between ligand atoms and protein residues, as is done for the initial pipeline in the optimization methodology. In this analysis, we first narrow our selection to the ten ligands with the most favorable binding affinities. Then, we select ligands that can be broken into fragments by BRICS that are in common with the fragments generated from the original source ligand database to facilitate analysis of how fragment conformation and binding may (or may not) be altered through the optimization process. We visualize the predicted ligand conformation and bonds using PyMOL, as shown below. To trace back the conformation of fragments within the ligands, we employ the BRICS algorithm used for fragmentation in the main pipeline to re-fragment the synthesized ligands. Critically, the optimization method is directed to improving binding efficacy (and any other optimization criteria) based on calculations that are made on the modified ligand structure, thereby accounting for any conformational changes. We focus on detailed analysis of the designed ligands with highly favorable binding affinity for the targets analyzed in the paper, TIPE2, RelA, and Spike RBD, which are shown in turn below.

Overall, we find that the fragments, even when disassociated from their original molecular context and integrated into new ligands, tend to adopt conformations within the binding pocket that are remarkably similar to their configurations in source ligands. Specifically, the case studies on receptor targets TIPE2 and RelA exemplify how fragments, despite their reintegration, align closely with their favorable binding positions observed in pre-identified ligands. These outcomes underscore the fragments' inherent propensity to revert to or maintain their effective binding conformations, thus validating our methodological approach against the concern that the loss of original 3D structure during fragmentation could compromise the ligand optimization process.

1. **TIPE2**

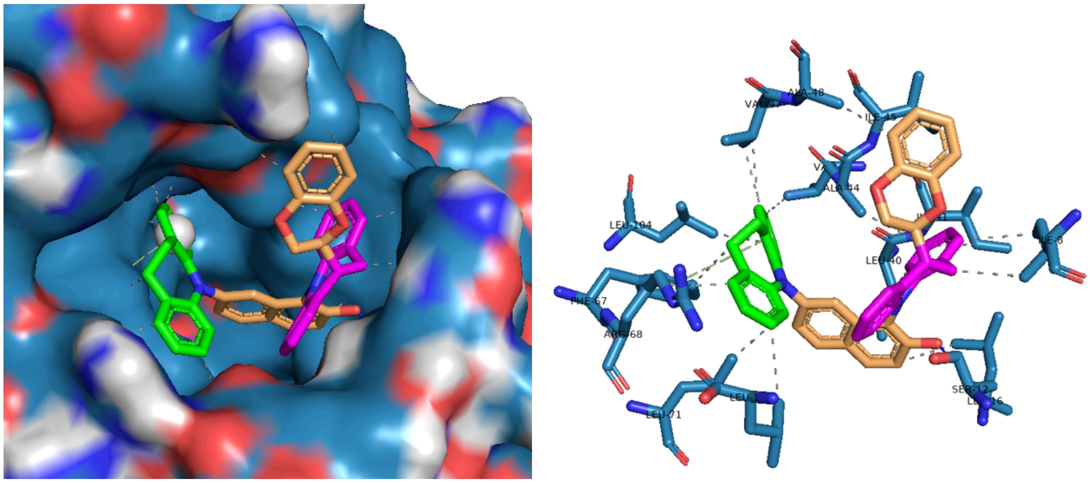

Supplementary Figure 4. PyMol diagrams showing one of the synthesized ligands (output from the second stage of optimization) predicted to have one of the ten most favorable binding affinities for TIPE2 and which also has fragments generated by BRICS in common with fragments in the source fragment database (i.e. generated by the initial FDSL-DD pipeline from a database of source ligands) to further analysis. The ligand is shown in the predicted conformation and position in the receptor binding pocket according to Autodock VINA (left) and the wire diagram shows predicted bonds between ligand atoms and receptor amino acids as generated by PLIP.

The ligand in Supplementary Figure 4 contains two fragments produced by BRICS that are colored in green and magenta with the following structure:

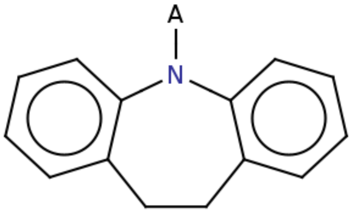

This fragment is present within 3 ligands in the original source dataset (the initial population of ligands used to create fragments through the FDSL-DD pipeline). The following two ligands in the "Source Ligand" column have similar conformations within the binding pocket as the generated ligand.

Color	Generated Ligand (same molecule, different view)	Source Ligand

Green	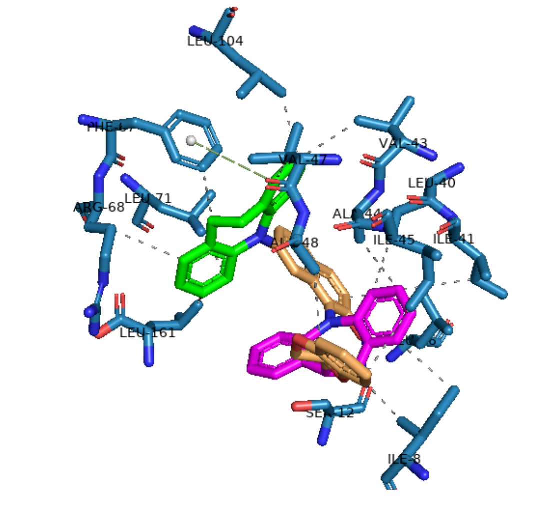	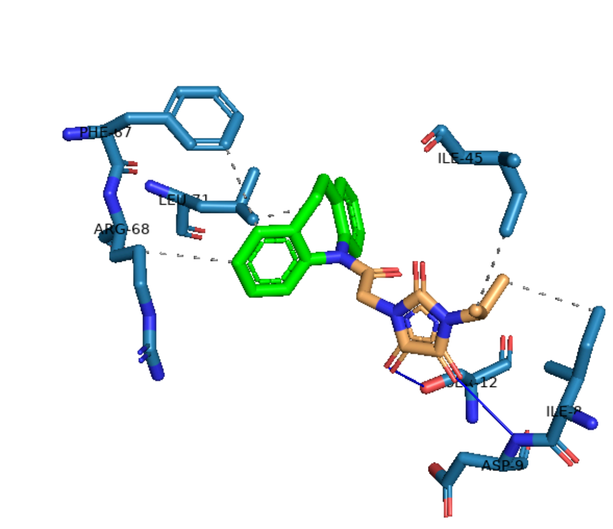
Magenta	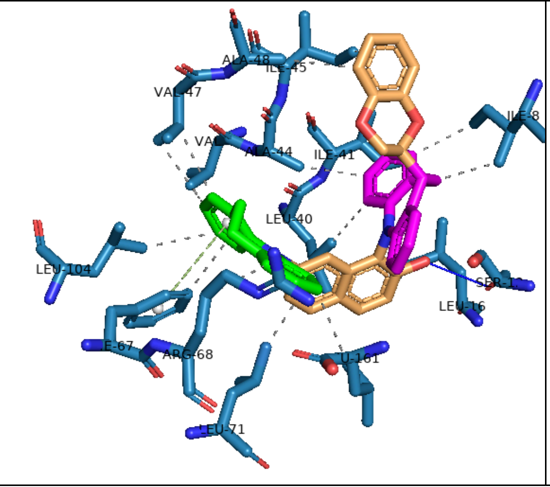	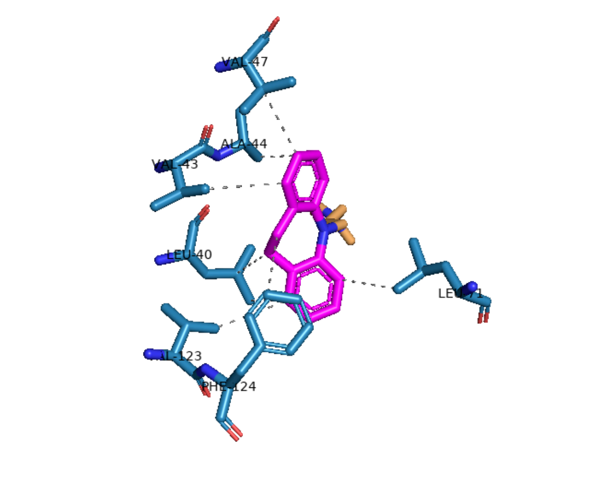

The images in the table above highlight the conformation of the ligand (left column) and the fragments it was likely sourced from (right column). As can be seen in the table, the conformations within the binding column are moderately conserved between the generated ligand and the fragments.

Notably, the images above are the raw outputs of PLIP (aside from coloring added later) and include exclusively amino acid interactions recognized by PLIP. Therefore, the binding pockets may appear different between the final ligand and the source ligands, as some amino acids may not interact with the respective ligand and thus not be included in an image. The table below summarizes the interactions between fragment substructures and amino acids in the binding pocket, referring to residue and ligand atom numbers that can be found in the PDB files output by Autodock VINA (mapped through the PLIP output).

**Fragment Color**	**Overlapping Amino Acids between generated ligand and fragments**	**Unique Residues (to generated ligand)**	**Unique Residues (to source ligand)**

Green	PHE67, ARG68, LEU71	LEU161, LEU104, VAL43, VAL47	None
Magenta	ALA44, LEU40	ILE8, ILE41	LEU71, VAL123, VAL 47, VAL 43, PHE 124

**Hydrophobic Interactions**					
**Index**	**Residue**	**AA**	**Distance**	**Ligand Atom**	**Protein Atom**
1	8	ILE	3.53	1260	8
2	8	ILE	3.17	1262	7
3	16	LEU	3.76	1231	67
4	40	LEU	3.6	1257	274
5	41	ILE	3.77	1258	280
6	43	VAL	3.5	1251	297
7	44	ALA	3.4	1258	303
8	45	ILE	3.66	1276	311
9	47	VAL	3.55	1251	327
10	47	VAL	3.41	1250	325
11	48	ALA	3.37	1274	332
12	67	PHE	3.49	1247	480
13	68	ARG	3.65	1245	487
14	71	LEU	3.46	1242	518
15	104	LEU	3.59	1252	770
16	161	LEU	3.47	1243	1227

**Hydrogen Bonds**									
**Index**	**Residue**	**AA**	**Distance H-A**	**Distance D-A**	**Donor Angle**	**Protein donor?**	**Side Chain**	**Donor Atom**	**Acceptor Atom**
1	12	SER	2.51	3.08	119.32	No	No	1229 [O3]	36 [O2]

**pi-Stacking**							
**Index**	**Residue**	**Residue**	**Distance**	**Angle**	**Offset**	**Stacking Type**	**Ligand Atoms**
1	67	PHE	5.14	69.34	0.95	T	1249, 1250, 1251, 1252, 1253, 1254

Because the fragments from the source pool end up in similar conformations, it is potentially arguable that the fragments tend to end up in their ideal conformations, even though the optimization process results in a loss of the original 3D structure. In addition, especially for the example above, because the fragments are relatively rare in the source ligand database (they occur only 3 times), the generated ligands were created to specifically utilize the binding potential of the fragments as they were found in source ligands, given that the fragment within the context of the synthesized ligand still ends up in similar binding regions as the source.

2. RelA

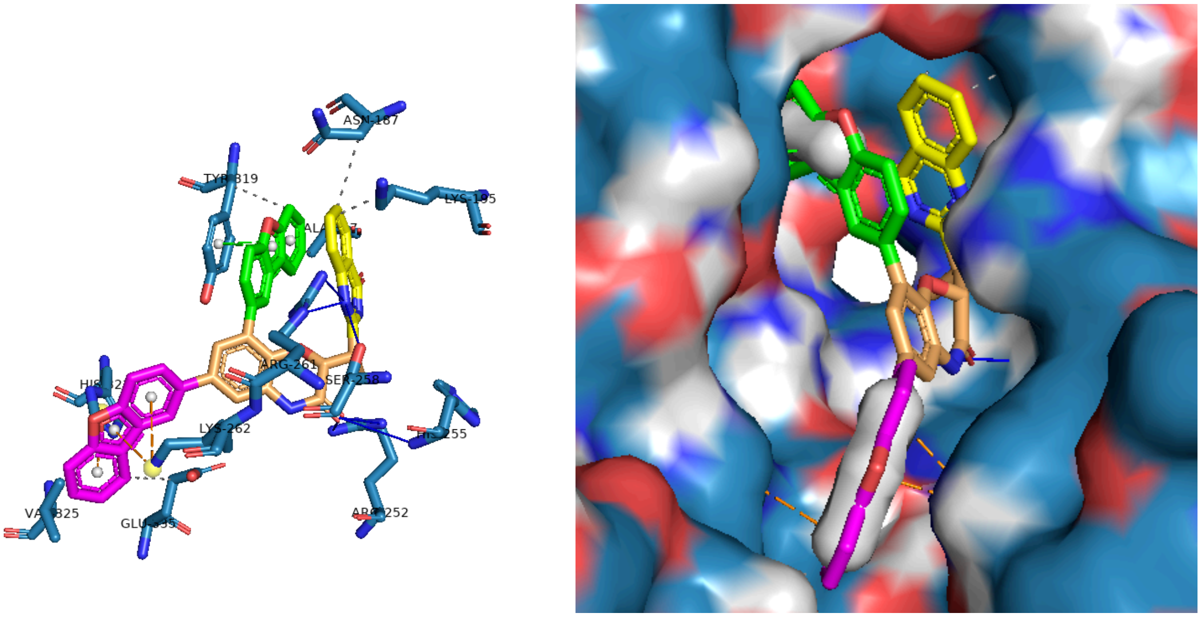

Supplementary Figure 5. PyMol diagrams showing one of the synthesized ligands (output from the second stage of optimization) predicted to have one of the most favorable binding affinities for RelA (selected to also have BRICS-generated fragments in common with the source ligand database). The ligand is shown in the predicted conformation and position in the receptor binding pocket according to Autodock VINA (left) and the wire diagram shows predicted bonds between ligand atoms and receptor amino acids as generated by PLIP.

After fragmentation via BRICS, the following two fragments in the ligand shown in Supplementary Fig. 5 were identified in the source pool of fragments:

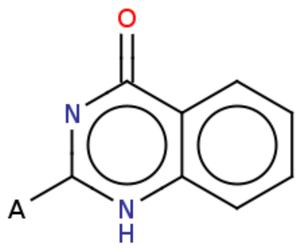

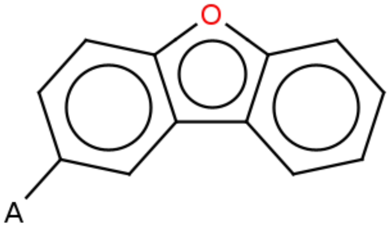

In Supplementary Fig. 5, the fragment shown on the left above is colored yellow, and the second on the right appears colored in green and magenta. Although these fragments were much more common in the source pools than the fragment identified for TIPE2, they are found to have similar conformations within the following source ligands.

Color	Generated Ligand (same molecule, different view)	Source Ligand
**Yellow**	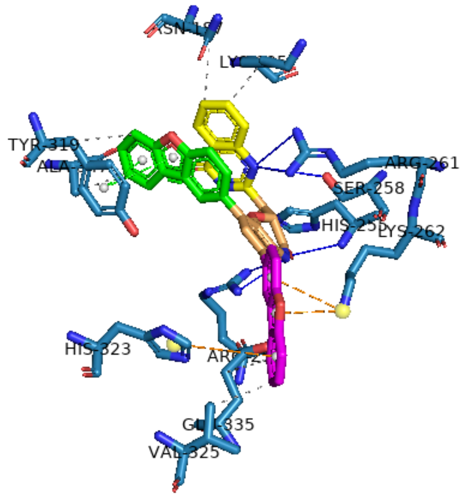	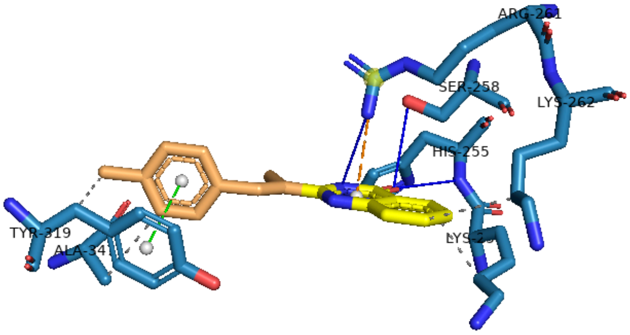

Green

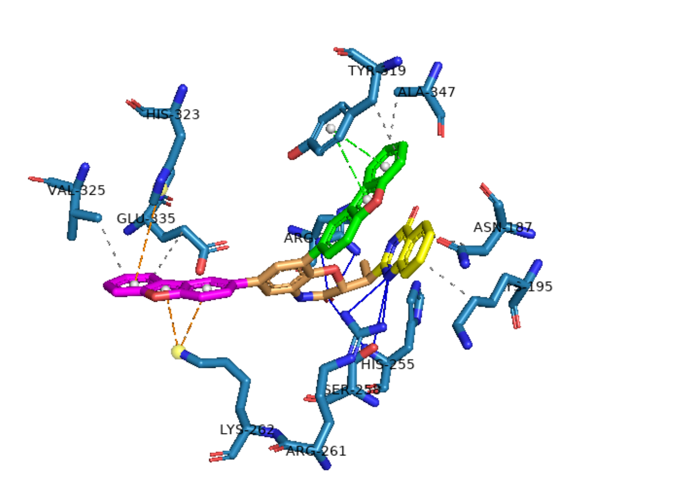

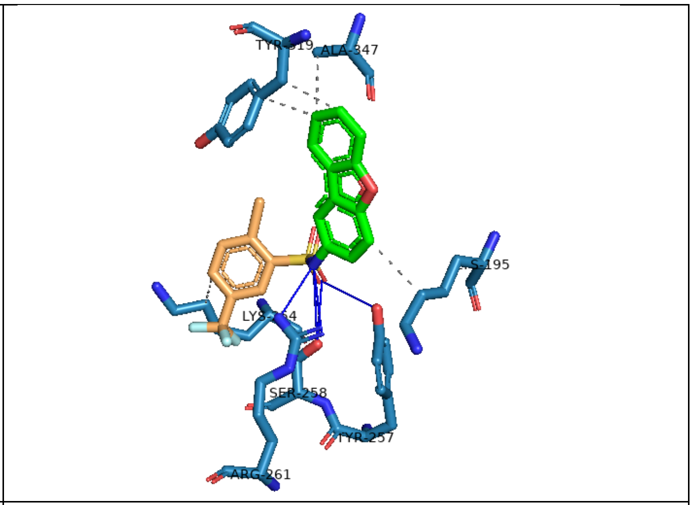

Magenta

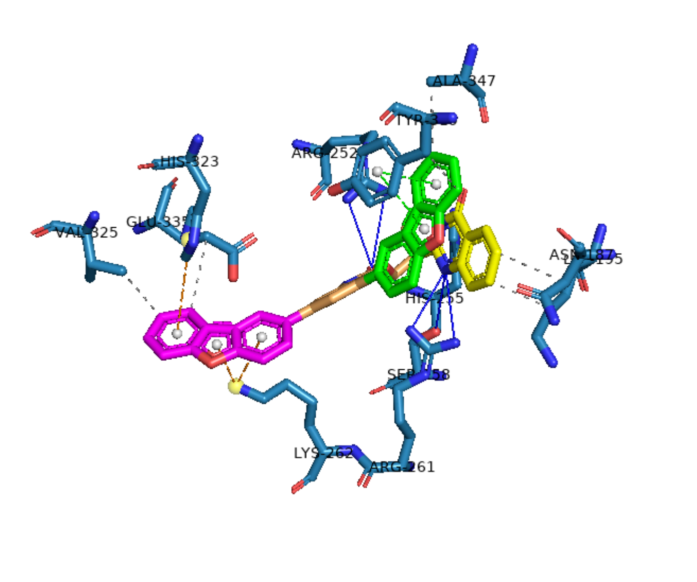

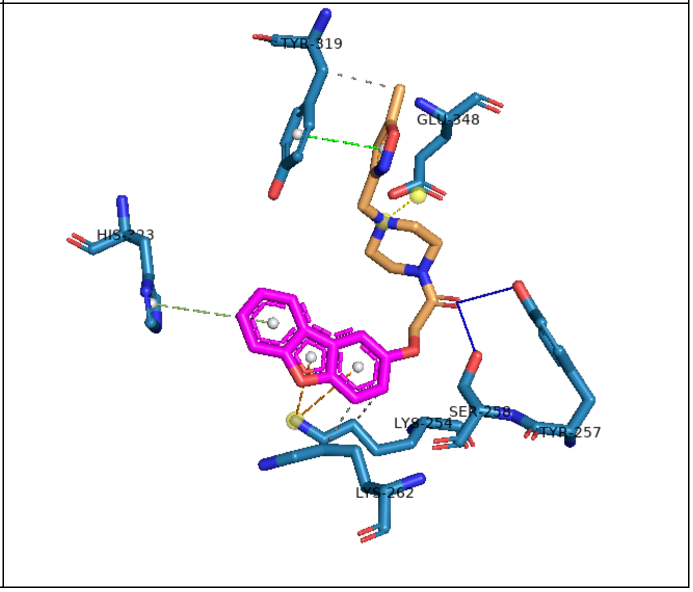

The green and magenta fragments have very similar positioning in the binding pocket between both generated ligand and source ligand. Although the yellow fragment in the above figure appears to express an extremely varied orientation within the binding pocket, it still exhibits similar hydrogen bonding interaction with SER258 and ARG251. The table below summarizes the similarities and differences between the fragment interactions and the binding pocket within the source ligands and generate ligands.

Fragment Color	Overlapping Amino Acids between generated ligand and fragments)	Unique Residues (to generated ligand)	Unique Residues (to source ligand)
**Yellow**	SER258, ARG261	LYS195, ASN187	LYS262, HIS255, LYS254
**Green**	ALA347, TYR319	None	LYS195
**Magenta**	LYS262, HIS323	VAL325, GLU335	LYS254

Hydrophobic Interactions					
Index	Residue	AA	Distance	Ligand Atom	Protein Atom
1	187	ASN	3.7	4838	1342
2	195	LYS	3.43	4839	1404
3	319	TYR	3.73	4823	2463
4	325	VAL	3.51	4812	2517
5	335	GLU	3.86	4813	2580
6	347	ALA	3.47	4825	2684

Hydrogen Bonds									
Index	Residue	AA	Distance H-A	Distance D-A	Donor Angle	Protein donor?	Side chain	Donor Atom	Acceptor Atom
1	252	ARG	2.68	3.6	156.58	Yes	Yes	1905 [Ng+]	4803 [O2]
2	252	ARG	3.16	3.98	141.72	Yes	Yes	1906 [Ng+]	4803 [O2]
3	255	HIS	2.89	3.74	145.53	Yes	No	1923 [Nam]	4803 [O2]
4	258	SER	3.08	3.84	136.23	Yes	Yes	1958 [O3]	4842 [Npl]
5	258	SER	2.93	3.84	153.01	No	Yes	4842 [Npl]	1958 [O3]
6	261	ARG	2.63	3.38	133.79	Yes	Yes	1991 [Ng+]	4842 [Npl]
7	261	ARG	2.91	3.62	129.5	Yes	Yes	1990 [Ng+]	4842 [Npl]

pi-Stacking							
Index	Residue	AA	Distance	Angle	Offset	Stacking Type	Ligand Atoms
1	319	TYR	4.05	4.95	1.98	P	4820, 4821, 4822, 4827, 4828
2	319	TYR	3.56	5	0.33	P	4822, 4823, 4824, 4825, 4826, 4827

pi-Cation Interactions							
Index	Residue	AA	Distance	Offset	Protein charged?	Ligand Group	Ligand Atoms

1	262	LYS	3.47	0.48	Yes	Aromatic	4807, 4808, 4809, 4814, 4815
2	262	LYS	3.89	1.8	Yes	Aromatic	4804, 4805, 4806, 4807, 4815, 4816
3	323	HIS	5.18	1.94	Yes	Aromatic	4809, 4810, 4811, 4812, 4813, 4814

Since these fragments were frequently seen in the source pool of fragments, we cannot assume that these specific conformations are the source of the fragments within the generated ligand. However, they do demonstrate that these fragments expressed similar confirmations in simpler molecules, indicating strong interactions within those regions for these particular substructures.

3. Spike RBD

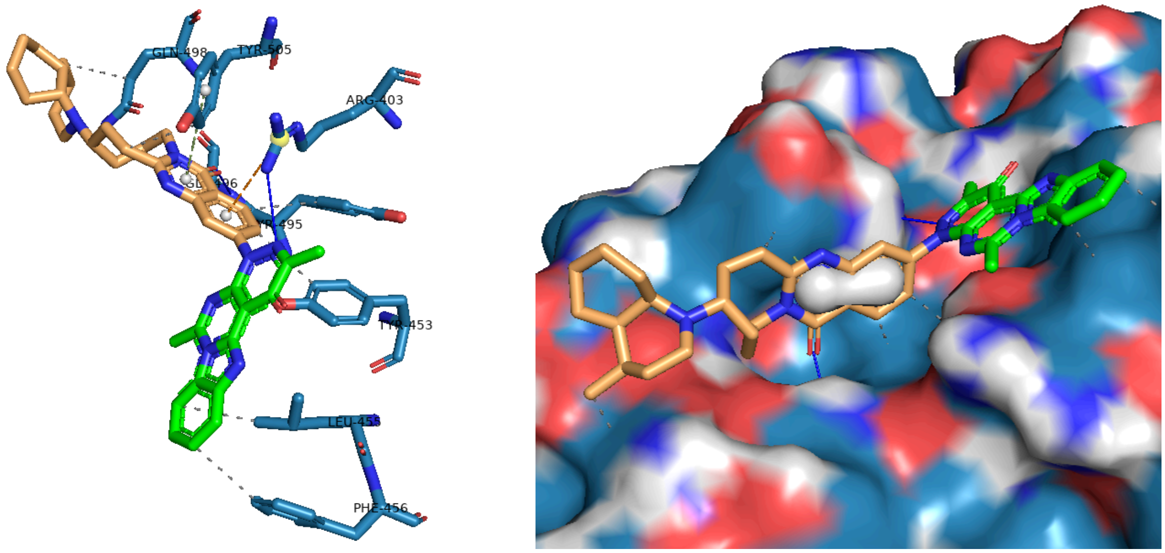

Supplementary Figure 6. PyMol diagrams showing one of the synthesized ligands (output from the second stage of optimization) predicted to have one of the more favorable binding affinities for Spike RBD; however, unlike the case for TIPE2, and RelA, the binding affinities tended to be lower. That said, as shown in the figures above, there was still favorable predicted binding within a much smaller binding pocket. The ligand is shown in the predicted conformation and position in the receptor binding pocket according to Autodock VINA (left) and the wire diagram shows predicted bonds between ligand atoms and receptor amino acids as generated by PLIP.

Notably, unlike TIPE2 and RelA as discussed above, there was not as much similarity between generated ligands and fragment pool, primarily because fragments with the best VINA scores did not produce fragments through BRICS present in source dataset. For example, the fragment that is colored green in Supplementary Fig. 6 is found in the fragment pool generated by the initial pipeline (i.e. in the source ligands). However, it is found in a different location relative to the predicted conformation in the synthesized ligand shown in Supplementary Fig. 6. The fragment in this conformation nonetheless hydrophobic interactions with PHE456 and LEU455 + hydrogen bonding with ARG403 and fits the pocket well in that region.

Indeed, while most favorable computationally synthesized ligands obtained through Spike RBD do not have the same conformation-retaining features as those for RelA and TIPE2 shown above, the Spike RBD target presents particularly challenging geometric constraints. The ligand shown in Supplementary Fig. 6 proves to have strong binding interactions with the pocket, despite the pocket's limited area for interaction compared to RelA and TIPE2.

**Hydrophobic Interactions**					
**Index**	**Residue**	**AA**	**Distance**	**Ligand Atom**	**Protein Atom**
1	453	TYR	3.89	1544	956
2	455	LEU	3.8	1570	978
3	456	PHE	3.93	1572	989
4	495	TYR	3.74	1545	1303
5	498	GLN	3.79	1584	1329
6	505	TYR	3.8	1554	1379

**Hydrogen Bonds**									
**Index**	**Residue**	**AA**	**Dist. H-A**	**Dist. D-A**	**Donor Angle**	**Protein donor?**	**Side chain**	**Donor Atom**	**Acceptor Atom**
1	403	ARG	2.78	3.25	109.62	Yes	Yes	566[Ng+]	1562 [Nar]
2	496	GLY	2.12	3.08	163.87	Yes	No	1309 [Nam]	1548 [02]

**pi-Stacking**							
**Index**	**Residue**	**AA Dist.**	**Angle**	**Offset**	**Stacking Type**	**Stacking Type**	**Ligand Atoms**
1	505	TYR	4.62	63.71	1.48	T	1546, 1547, 1549, 1550, 1551, 1552

**pi-Cation Interactions**

Index	Residue	AA	Distance	Offset	Protein charged?	Ligand Group	Ligand Atoms
1	403	ARG	4.77	0.85	Yes	Aromatic	1543, 1544, 1545, 1546, 1552, 1553
